# The Immunogenic Potential of Recurrent Cancer Drug Resistance Mutations: An *In Silico* Study

**DOI:** 10.3389/fimmu.2020.524968

**Published:** 2020-10-08

**Authors:** Marco Punta, Victoria A. Jennings, Alan A. Melcher, Stefano Lise

**Affiliations:** ^1^Centre for Evolution and Cancer, The Institute of Cancer Research, London, United Kingdom; ^2^Department of Immunity and Infection, Leeds Institute of Medical Research, Leeds, United Kingdom; ^3^Division of Radiotherapy and Imaging, The Institute of Cancer Research, London, United Kingdom

**Keywords:** immunogenomics, cancer vaccines, cancer targeted therapy, tumor neoantigen predictions, drug resistance, resistance mutations

## Abstract

Cancer somatic mutations have been identified as a source of antigens that can be targeted by cancer immunotherapy. In this work, expanding on previous studies, we analyze the HLA-presentation properties of mutations that are known to drive resistance to cancer targeted-therapies. We survey a large dataset of mutations that confer resistance to different drugs and occur in numerous genes and tumor types. We show that a significant number of them are predicted *in silico* to be potentially immunogenic across a large proportion of the human population. Further, by analyzing a cohort of patients carrying a small subset of these resistance mutations, we provide evidence that what is observed in the general population may be indicative of the mutations’ immunogenic potential in resistant patients. Two of the mutations in our dataset had previously been experimentally validated by others and it was confirmed that some of their associated neopeptides elicit T-cell responses *in vitro*. The identification of potent cancer-specific antigens can be instrumental for developing more effective immunotherapies. In this work, we propose a novel list of drug-resistance mutations, several of which are recurrent, that could be of particular interest in the context of off-the-shelf precision immunotherapies such as therapeutic cancer vaccines.

## Introduction

Cancer cells express a typically aberrant protein repertoire compared to that of normal cells. These aberrations, whether functional (drivers) or non-functional (passengers), have the potential to generate peptide antigens that are not (or are only partially) subjected to central or peripheral tolerance. As such, when presented by human leukocyte antigen (HLA) complexes on the surface of cancer cells, these antigens might lead to recognition by cytotoxic T-cells and, eventually, to immunomediated tumor clearance (1, 2). Tumors, however, can develop sophisticated escape mechanisms (3) and immune evasion is now recognized as one of the hallmarks of cancer (4).

Cancer immunotherapies seek to restore the ability of the host’s immune system to recognize tumor antigens and attack the cells that express them (5). Checkpoint blockade therapies (CBTs), in particular, act by inhibiting negative immune-checkpoint receptors and thus reinvigorating the cytolytic activity of the patient’s T-cell repertoire (6). As mentioned above, cancer cells that present an aberrant immunopeptidome are most likely to be targeted by T-cells. Despite remarkable successes, CBTs are to date approved for treatment in a limited number of solid malignancies, with only a fraction of patients responding (7). As great efforts are being made toward improving the scope and efficacy of CBTs (8), there is growing interest for the identification of patients’ specific, highly immunogenic antigens that could be used for more targeted treatments (9), possibly in combination with CBTs. These include therapeutic cancer vaccines (10–12) that can be produced *ex vivo* and delivered to the patient in the form of peptides, peptide-encoding RNA/DNA molecules, or using peptide-loaded autologous dendritic cells or viruses (13). Identification of tumor antigens that can serve for these purposes is thus a priority (14, 15).

Historically, peptides belonging to a normal cell proteome but preferentially or almost exclusively expressed in cancer cells (“tumor-associated antigens” or TAAs) were the first to be targeted for the clinic (11, 16, 17) along with oncoviral antigens (encoded by oncogenic viruses) (18). Although the clinical development of vaccination strategies against TAAs continues, they are now generally regarded as less-than-ideal and often weak effectors, primarily because of incomplete tumor specificity and partial central tolerance (13, 19). Increasingly, researchers are focusing their attention on cancer-specific peptides such as those associated with passenger mutations (10, 20–26), somatic gene fusions (27), aberrantly expressed tumor transcripts (28) or tumor-specific alternatively spliced isoforms (29) and post-translational modifications (30, 31).

In this study, building on previous works (32–34), we present a comprehensive *in silico* survey of the antigenic potential of peptides associated with cancer drug resistance mutations. Resistance mutations emerge in the context of targeted therapies, which are aimed at tumors that depend for their growth on specific oncogenes (35). This addiction makes such tumors vulnerable, at least in principle, to drugs that inhibit the relevant protein(s). Targeted therapies are available for an increasing number of hematological and solid malignancies [e.g (36–38)], however, a significant fraction of patients either don’t respond to treatment or eventually relapse. Intrinsic (germline or somatic) and acquired (somatic) resistance is mediated by a range of different molecular mechanisms (39). Among them is the pre-existence (possibly, if somatic, at very low allele frequencies) or the acquisition following treatment of protein-modifying mutations on the targeted oncogenes or on other genes in the same or alternative pathways (40, 41).

Resistance mutations possess a number of properties that are appealing in the context of precision immunotherapy: they are tumor-specific, thus generating neoantigens that are less likely to be subjected to central or peripheral tolerance or to elicit an autoimmune response (42); because they drive resistance, they are expected to be specifically expressed in therapy-resistant clones; they are usually found on oncogenes, hence making therapy-escape by the tumor through their down-regulation harder; and, finally, several of them are known to recur in different patients (i.e., they are not patient-specific) making them potential targets for developing off-the-shelf rather than fully personalized and potentially highly expensive precision therapies (43). Here, we report on 226 resistance mutations (source: COSMIC) that pertain to numerous genes, tumor types, and drugs and we study their potential immunogenicity in relation to a set of 1,261 individuals from the 1000 Genomes (1000G) project encompassing a landscape of 194 HLA-A, -B, and -C class I allotypes. By analyzing *in silico* their HLA class I presentation properties, as well as those of their associated wild type peptides, we show that several of these mutations generate neopeptides that are predicted to have immunogenic potential across a significant fraction of individuals in our 1000G dataset. Further, we investigate a cohort of 92 patients from the Hartwig Medical Foundation database (44), carrying a small subset of these resistance mutations (four in total). Our analysis indicates that in a fraction of these patients the neopeptides associated to the mutations are predicted to be potentially immunogenic and, when set against a backdrop of control patients, we see no evidence that the mutations undergo negative selection by the immune system. Also, comparison with HLA-presentation properties of these four mutations in the 1000G population suggests that estimates based on the latter can be indicative of the immunogenic potential of the wider set of COSMIC mutations in resistant patients. In the context of previous publications that showed how neopeptides from two resistance mutations [E255K in BCR-ABL1 (32) and T790M in EGFR (33, 34)] could elicit T-cell responses *in vitro*, our results support the idea that drug resistance mutations might be an important (and, in time, potentially expanding) source of tumor antigens for precision immunotherapies. In particular, this opens up the possibility of tracking the development of recurrent resistance mutations (for example, in circulating tumor DNA), while patients are treated on a particular drug, and using an off-the-shelf vaccine targeting the relevant resistance neoantigen to prolong the period of clinical benefit.

## Methods

### Mutation Datasets

We downloaded directly from Marty et al. (45). the following mutation datasets: passenger mutations (1,000 in total), recurrent mutations (1,000), germline single nucleotide polymorphisms (SNPs) (1,000), random mutations (3,000). In Marty et al., recurrent and passenger mutation lists are derived from The Cancer Genome Atlas (TCGA) data (46). In particular, recurrent mutations, which we will refer to as driver mutations here, are defined as those found within a list of 200 tumor-associated genes (47) and observed in at least three TCGA samples. All somatic TCGA mutations not occurring in the list of tumor-associated genes are considered as passengers. Germline SNPs are common germline variants that are sampled from the Exome Variant Server. Finally, random mutations are generated randomly in human proteins from Ensembl (release 89; GRCh37). From the initial list of 1,000 driver mutations, we further extracted those that are observed in at least 30 TCGA patients and we labeled them as *highly recurrent (HR) driver mutations* (32 in total).

Our list of resistance mutations was obtained from the CosmicResistanceMutations.tsv file that we downloaded from the COSMIC website (COSMIC version 86). From this initial list, we manually removed a few entries (COSM5855836, COSM1731743, COSM5855814, COSM3534174, COSM763) that appeared to be duplicates of other entries (COSM5855837, COSM1731742, COSM5855815, COSM3534173, COSM125370, respectively), those for which information about the exact amino acid substitution was not provided in COSMIC and non-missense mutations. Overall, we obtained 226 resistance mutations (**Supplementary Table 1**). Note that four of them also appeared in our list of 32 HR driver mutations (NRAS Q61R and Q61K, PIK3CA E545K, BRAF V600E), while 11 in total overlapped with the full list of driver mutations. Genes, tissues, tumor subtypes, and drugs to which these mutations are associated are reported in **Supplementary Table 2**. In the file from COSMIC, each mutation is listed as many times as the number of patients in which it has been reported in the scientific literature. Although this can provide us with valuable information on the prevalence of different mutations in patients with a specific tumor type that have been treated with the same drug, comparisons across different tumor types, genes, and drugs are more complicated. Indeed, the number of cases reported in COSMIC can be influenced by several factors, including a tumor’s incidence or the time that has passed since a drug’s approval. For example the EGFR C797S mutation, a mutation of particular clinical relevance conferring resistance to the lung carcinoma third-generation EGFR inhibitor osimertinib (48), is reported in COSMIC to have occurred in nine patients. These are much fewer, for example, than the 487 records for the T790M mutation. Osimertinib, however, is a relatively recent drug (FDA-approved in 2015) when compared to some of the drugs T790M confers resistance to (e.g., Gefitinib, FDA-approved in 2003). Here, notwithstanding these limitations, we made use of the number of occurrences in COSMIC to obtain at least a rough separation between rare and more frequent resistance mutations, with the latter being the ones that were more likely to be relevant in the context of off-the-shelf cancer vaccine development. In particular, we defined a set of *highly recurrent (HR)* resistance mutations (at least 20 patients in COSMIC, 22 mutations in total) (**Supplementary Table 1**). Yet another two subsets of resistance mutations we used were “resistance-BCR-ABL1” and “resistance-no-BCR-ABL1,” which contain only COSMIC resistance mutations found or not found in BCR-ABL1, respectively (102 and 124 in total, **Supplementary Table 1**). Note that although patients reported to have multiple resistance mutations might carry compound mutations that could be generating multiple-mutant neopeptides, this type of information is generally not available from COSMIC (zygosity is also for the most part unknown); as a consequence, we considered all resistance mutations we selected as being “isolated” mutations.

### Generation of Mutation-Associated Peptides

In order to calculate the HLA-presentation likelihood for the peptides generated by the above sets of mutations, we needed to map each mutation to a protein sequence. Here, we used human protein sequences from EnsEMBL as found in the Homo_sapiens.GRCh37.pep.all.fa.gz file (downloaded from EnsEMBL and, hereafter, referred to as “EnsEMBL protein file”). For resistance mutations, we obtained from the CosmicResistanceMutations.tsv table the EnsEMBL transcript ids, all of which had a corresponding protein entry in the EnsEMBL protein file. For all other sets of mutations, we first extracted the gene id from table S3 of (45); then, we generated (Jan 2018) from the UCSC Genome Table Browser the mapping between gene ids [Human Genome Organization Gene Nomenclature Committee (HGNC) symbols] and canonical EnsEMBL transcripts and, additionally, from the EnsEMBL BioMart the mapping between gene ids and non-canonical EnsEMBL transcripts. Finally, given a mutation and its associated gene id, we tried to map the mutation to the EnsEMBL canonical transcript sequence for that gene id. If we were not successful, we tried to map the mutation to a non-canonical transcript sequence for the same gene id. If even in this second case we could not find any appropriate mapping, we discarded the mutation. For mutations that we were able to map to a transcript, we could then find the corresponding protein sequence in the EnsEMBL protein file. According to this protocol, occasionally, two different mutations found in the same gene could end up being mapped to two different EnsEMBL transcripts and hence protein sequences. We only considered missense mutations (in particular, single amino acid substitutions); we did not consider indels. The final mutation count for each set was as follows: 961 passengers, 999 drivers (32 of which constitute our HR drivers list), 970 germline SNPs, and 2,758 random mutations, along with the 226 resistance mutations already mentioned above. These final lists of mutations are reported in **Supplementary Table 1** together with their Population-Wide Median Harmonic-Mean Best Rank (PMHBR) score (when using all three HLA genes for calculating the score, see below). The ids of the EnsEMBL transcripts used for genes in these datasets are shown in **Supplementary Table 3**.

For each mutation part of the datasets in **Supplementary Table 1**, we used an in-house Python script to generate all possible peptides of length 8 to 11 that spanned the mutation. For mutations that did not fall within the first 10 or last 10 positions of a transcript this meant generating a total of 38 peptides (or correspondingly less otherwise). A wild type peptide associated to a specific mutant peptide is identical to the mutant peptide except for the fact that the mutated amino acid is reverted to the wild type one.

### Datasets of Healthy Individuals and Patients With Known HLA Allotypes

We obtained a list of 1000G healthy individuals with their associated HLA class I allotypes from ftp://ftp.1000genomes.ebi.ac.uk/vol1/ftp/technical/working/20140725_hla_genotypes/20140702_hla_diversity.txt. This dataset included 1,267 unique individuals from the 1000 Genomes Project, covering 14 populations and four major ancestral groups (49). Each individual was annotated with their six HLA class I allotypes. However, in several cases each of the six allotypes were represented by multiple entries. These typing ambiguities reflect allotypes that do not differ on exons 2 and 3 of the HLA gene, that is, the exons carrying the antigen recognition sites. In these cases, we considered only the first reported entry for each allotype. We excluded individuals that had allotypes that were not well defined at the four digit level or that were not present in the NetMHCpan-4.0 library of HLA allotypes (NetMHCpan-4.0 is the method that we use for predicting HLA-presentation, see below), these were: HLA-A03:03N, HLA-B44, HLA-C15, HLA-C14XX, HLA-C0140, and those labeled “0000.” The complete list of 1,261 individuals and associated allotypes that we used is given in **Supplementary Table 4**. In the following, we refer to this as the *1000G dataset* and use it to represent the HLA class I haplotypes that we expect to find in individuals within the general population. We additionally used a list of haplotypes from 7,726 TCGA patients obtained from the file “Shukla_Wu_Getz_Polysolver_HLA_Types_2015.tsv” (50) and deposited under controlled access in dbGAP (accession code: phs000178). Note that from the original list of 7,727 haplotypes in the file, we removed the haplotype of one of the patients with an HLA allotype, HLA-A*01:04N, for which NetMHCpan-4.0 could not predict peptide binding likelihoods.

### The Hartwig Medical Foundation Database: Extraction of a Subset of Patients With COSMIC-Annotated Resistance Mutations

The Hartwig Medical Foundation Database (HMFD) (44) is a large resource of metastatic tumor data, generated through whole genome sequencing and matched with detailed clinical information (https://www.hartwigmedicalfoundation.nl/en/database/). We obtained access to the HMFD and downloaded the *vcf* files containing the somatic variant calls for 4,479 patients. In these samples, we looked for COSMIC resistance entries for which we had knowledge of the exact genomic position of the single nucleotide substitution leading to the missense mutation (124 total out of 226 mutations in COSMIC). For us to consider a resistance mutation called in an HMFD sample, the variant had to have a PASS flag and occur on the same transcript on which it was annotated in COSMIC. We found 238 patients that carried a resistance mutation that satisfied all of the above criteria and when discarding samples with multiple such mutations. Minimum, median, and maximum variant allele frequencies for the 238 mutations in these patients were 3.7, 25.6, and 96.8%, respectively. From this list of 238 samples we further removed cases that in HMFD had no associated metadata specifying pre-biopsy treatments and/or had a tumor tissue type that did not match the one reported in COSMIC for the same resistance mutation and/or were not first-biopsy samples or, finally, for which the downloaded cram file was not labeled as deduplicated. This left us with a list of 127 samples spanning a set of 21 different COSMIC resistance mutations in total. For our analysis, we finally selected the four mutations with the highest count in this list, namely: D538G in ESR1 (42 samples), Y537S in ESR1 (26 samples), T790M in EGFR (13 samples), and T878A in AR (11 samples) for a total of 92 samples. These four mutations covered three different tumor types: breast (D538G and Y537S in ESR1), lung (T790M in EGFR), and prostate (T878A in AR). Although most patients for whom we considered samples underwent several treatments before the first biopsy, we checked that at least one of the treatments had been associated to the respective resistance mutation in the literature (51–53). All samples with D538G in ESR1 and all but one with Y537S in ESR1 belonged to patients that had pre-biopsy “aromatase inhibitor” treatment; the only patient with the Y537S ESR1 mutation that had no aromatase inhibitor treatment had undergone tamoxifen hormonal therapy pre-biopsy. All 13 samples with T790M in EGFR belonged to patients that underwent “anti-EGFR” treatment pre-biopsy. All 11 samples with T878A in AR belonged to patients that underwent “anti-AR” treatment pre-biopsy. Note that these four mutations are also reported by COSMIC to commonly occur; in particular, COSMIC reports the presence of D538G and Y537S ESR1 in 35 and 22 patients, respectively, T790M EGFR in 487 patients and T878A AR in 17 patients. For comparison purposes, we additionally downloaded 35, 25, and 34 samples, respectively, for breast, lung, and prostate tumors. These samples were such that their *vcf* files did not feature any of the 226 COSMIC mutations with a PASS flag (this time, we looked for the matching amino acid substitution irrespective of the genomic position at which the mutation occurred) and were such that the patients they belonged to had undergone pre-biopsy treatments involving aromatase inhibitors (breast), anti-EGFR (lung) and anti-AR (prostate). Note that, although we initially aimed to collect 35 samples for each tumor tissue, we could not find more than 26 in lung satisfying the above criteria and, additionally, one sample each for lung and prostate was discarded after download because the cram file was not labeled as deduplicated. Further, we identified candidate driver mutations in the 186 HMFD samples under study (spanning both the ones carrying resistance mutations and the controls) from the list of TCGA recurrent mutations defined in Marty et al. (45). We identified a total of 65 different driver mutations in 121 samples; of these, 13 (in 55 samples) overlapped with the *highly recurrent* set in TCGA, i.e., present in at least 30 TCGA patients. Finally, we picked randomly 1,000 passenger mutations found in the selected HMFD samples as missense mutations that had a PASS flag in the vcf files and were not found in genes where we had a driver (Marty et al) or resistance (COSMIC) mutation. Of these, 917 were left after mapping gene ids to EnsEMBL transcripts (see above); their associated neopeptides were generated as described above.

For all of the 92 resistant patients and 94 control patients we considered, we predicted HLA class I haplotypes with the program POLYSOLVER [(v1.0) (50)] using their associated whole-genome sequencing (WGS) germline samples [median depth of sequencing around 38x (44)]. In particular, we ran the shell_call_hla_type script, with the following parameters: race = unknown, includeFreq = 1, and insertCalc = 0. Note that although POLYSOLVER was originally developed to run on whole exome sequencing data, benchmarking experiments indicate that it performs well also on WGS data at depth of sequencing 30x or higher (54–56).

We report the list of HLA haplotypes and HMFD patients’ ids for all HMFD samples we considered together with either the resistance mutation we found in their associated patients or, for the controls, the tumor tissue type in **Supplementary Table 5**.

### HLA-Presentation Scores

All HLA-presentation scores that we describe in the following are defined starting from eluted ligand likelihood percentile ranks of peptides with respect to HLA allotypes; these rank scores are obtained from the NetMHCpan-4.0 prediction method (57).

#### BR: Best Rank Score of a Mutation

Each missense mutation is associated to a set of (maximum 38) mutated peptides (see *Generation of Mutation-Associated Peptides* above). For each peptide in this set, we used the program NetMHCpan-4.0 (57) to calculate the eluted ligand likelihood percentile rank and predict the interaction core peptide (*Icore*) with respect to all HLA allotypes observed in our datasets (see above). The elution rank takes values in the range from 0 to 100, with lower values representing higher presentation likelihoods. The *Icore* is the part of the original peptide predicted by NetMHCpan-4.0 to be located in the HLA binding site, thus the peptide most likely to interact with the T-cells. In some of the cases in which the *Icore* is shorter than the original peptide, it may not span the mutation at all and may thus be equivalent to a wild type peptide. We defined the presentation score of a mutation with respect to a specific HLA allotype as the minimum elution rank among all associated peptides [this is the same as the “best rank” score used in (45)] but excluding those with a wild type Icore. We called this presentation score *BR*.

#### PMHBR: Population-Wide Median Harmonic-Mean Best Rank Score of a Mutation

In Marty et al. (45), the authors define a patient-specific presentation score for a mutation by using a harmonic mean to combine the six best rank scores of the patient’s six HLA allotypes (Patient Harmonic-Mean Best Rank or PHBR, see below). Unfortunately, COSMIC does not contain information about the HLA allotypes of the patients that develop specific resistance mutations. As a consequence, in order to provide an equal-ground comparison between all groups of mutations, we alternatively defined a score that is representative of the presentation properties of a mutation across a whole population. We calculated our Population-Wide Median Harmonic-Mean Best Rank (PMHBR) for a mutation *m* as:

(1) $$PMHBR\left(m\right)=median_{\left\{1000G\right\}or\;\left\{TCGA\right\}}\left(\frac{6}{\sum_{i=1}^{6}\frac{1}{BR_{i}\left(m\right)}}\right)$$

where the internal summation is taken over all six HLA allotypes of a given individual or patient (two each for HLA-A, -B, and -C) and the median is taken over the full set of 1,261 individuals in the 1000 Genomes Project dataset or, alternatively, the full set of 7,726 patients in TCGA. In other words, PMHBR is the median of the PHBR scores (see below) of a mutation calculated over a set of individuals or patients (note that also Marty et al. (45) define a median PHBR score although they calculate it for groups of mutations).

Lower PMHBR scores correspond to higher likelihoods for the mutation to be presented across our 1000G or TCGA populations. Because HLA-C proteins are generally expressed at lower levels with respect to HLA-A and HLA-B (58), in the *Supplementary Materials* we also report analyses in which the two HLA-C allotypes were omitted from the calculation of the harmonic average in (1).

#### IBR/PBR: Individual/Patient Best Rank Score

We additionally defined individual’s and patient’s best ranks (IBR and PBR, respectively) for a mutation *m* as the minimum BR of the mutation when considering all of the HLA allotypes of the individual, that is:

(2) $$IBR/PBR\left(m\right)=min_{individual^{\prime }s/patient^{\prime }s\;HLAs}\left(\;BR\right)$$

Note that with “individuals,” throughout this work, we always refer to members of the 1000G healthy population, while “patients” can be those found in TCGA or in the HMFD. IBR and PBR were useful for calculating the percentage of individuals or patients in which a mutation was likely to be presented according to a pre-defined threshold. For example, we could calculate the percentage of individuals or patients for which IBR(m) or PBR(m), respectively, were <0.5 or, alternatively, <2.0 (see *Results*).

#### IHBR/PHBR: Individual/Patient Harmonic Mean Best Rank Score

These are patient-specific harmonic average scores that we use in the analysis of passenger, driver, and resistance mutations in HMFD patients and are defined exactly as in Marty et al. (45). In particular, for a mutation *m*:

(3) $$IHBR/PHBR\left(m\right)=\;\frac{6}{\sum_{i=1}^{6}\frac{1}{BR_{i}\left(m\right)}}$$

where the internal summation is taken over all six HLA allotypes (two each for HLA-A, -B, and -C) of a given individual (in the 1000G dataset, IHBR) or patient (in the HMFD dataset, PHBR).

#### Comparison Between Mutant and Wild Type Peptide HLA-Presentation Scores

Given a mutation and an individual (or patient) with their associated HLA allotypes, we compared the individual’s (patient’s) HLA-presentation scores of mutant *versus* wild type peptides in the following ways. We first calculated the minimum eluted ligand likelihood percentile rank scores across all of the individual’s (patient’s) HLA allotypes for each pair of mutant *Icore* and corresponding wild type peptide generated by the mutation (we called these the mutant and wild type peptide MinRank, respectively; note that the MinRank is a property of the single peptide rather than of the mutation like the previously defined BR). We then did one of two things: i) we asked that at least one pair existed such that the MinRank of the mutant peptide was lower than a given threshold and the MinRank of the wild type peptide was equal or higher than the same or different (higher) threshold or ii) we asked that at least one pair existed such that the MinRank of the mutant peptide was lower than a given threshold and, additionally, lower than the one of the wild type peptide. In both cases, we used thresholds of 0.5 or 2.0. Indeed, NetMHCpan-4.0 eluted ligand likelihood percentile rank score values below 0.5 are usually said to indicate high presentation likelihood, values between 0.5 and 2.0 to indicate low presentation likelihood and values >2.0 to indicate that a peptide is not likely to be presented. We additionally used MinRank scores for the analysis of the immunogenic potential of individual peptides (rather than mutations) in the general population.

### Statistical Analysis and Plots

Throughout this study, statistical analysis was performed and plots were drawn using GraphPad Prism version 8.1.2 for OS X, GraphPad Software, La Jolla California USA, www.graphpad.com. In particular, to calculate multiple comparison-adjusted p-values we performed Kruskal-Wallis tests and Dunn’s *post hoc* tests; to calculate p-values of pair-wise comparisons we performed two-tailed Mann-Whitney tests; to calculate p-values for paired groups’ comparisons we performed two-tailed Wilcoxon tests.

## Results

**Figure 1:**
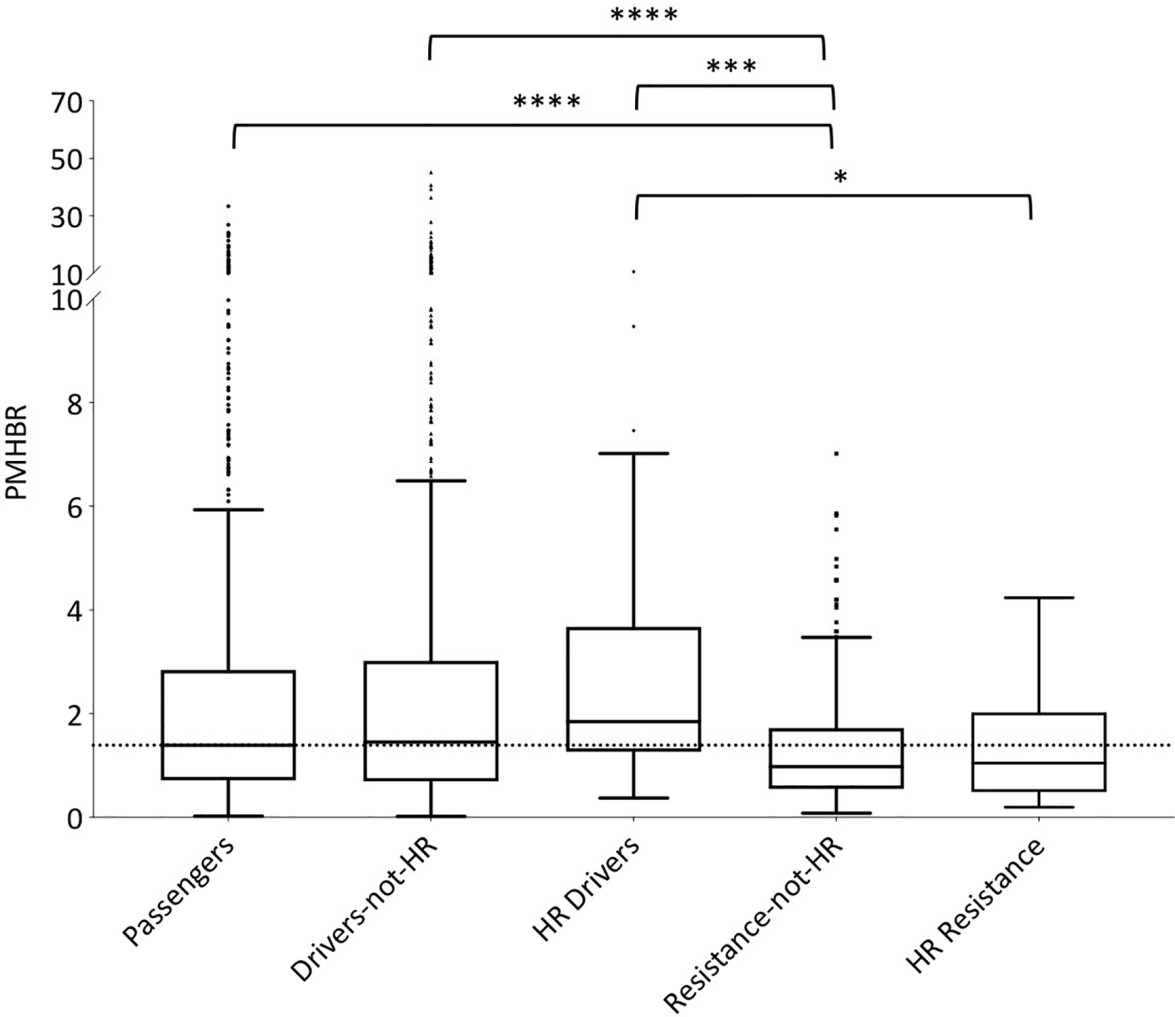
Distribution of PMHBR scores for different sets of mutations. Lower PMHBR values correspond to a higher likelihood of being presented by HLA class I complexes across our set of 1000G healthy individuals. We consider passenger and driver mutations from TCGA and resistance mutations from COSMIC. Driver mutations are split according to their level of recurrence in TCGA patients (drivers-not-HR = less than 30 patients, HR-drivers = at least 30 patients). Similarly, resistance mutations are split according to their level of recurrence in patients as reported in COSMIC (resistance-not-HR = less than 20 patients, HR-resistance = at least 20 patients). Note that absolute numbers in the level of recurrence for driver versus resistance mutations are not easily compared (see *Methods*). The dotted horizontal line is a guide for the eye and corresponds to the value of the median of the distribution for passenger mutations. Note that, for the sake of readability, the part of the y-axis corresponding to values of PMHBR above 10 is compressed. Asterisks indicate significance of differences between PMHBR score distributions calculated using a Kruskal-Wallis test followed by Dunn’s *post hoc* test. p-values are adjusted for multiple testing (all *vs.* all). (*) stands for p-value < 0.05, (***) for p-value < 0.001 and (****) for p-value < 0.0001. The lower and higher edges of each Tukey box represent the 25 and 75% percentile value, respectively. The horizontal line inside each box represents the median value.

In order for protein peptides to be immunogenic, it is necessary for them to be presented by HLA class I complexes and, additionally, to be able to escape central and peripheral tolerance. We start by comparing predicted HLA class I presentation scores of resistance-mutation associated neopeptides to those of neopeptides of different origin, across the general population (**Figure 1** and, additionally, **Supplementary Figure 1** for a violin plot of the same data). We use healthy individuals from the 1000 Genome project as representatives of the general population and the PMHBR defined in *Methods* as a HLA-presentation score. We can see that passenger and driver mutations that are not highly recurrent feature similar PMHBR score distributions (the same is true for germline SNPs and random mutations, **Supplementary Figure 2**). However, if we consider highly recurrent drivers (HR drivers, *Methods*) we see that their distribution of PMHBR scores is shifted toward higher values, indicating a lower likelihood of the associated neopeptides to be HLA-presented in the general population. Although these differences are not significant, the trend is in line with the observations made by Marty et al. that highly recurrent oncogenic mutations have universally poor HLA class I presentation and that a driver mutation’s frequency in cancer patients negatively correlates with the population ability to present it (45). If we now look at resistance mutations, we see that their PMHBR scores are generally lower than the ones of both passenger and driver mutations and that this appears to be independent on their level of recurrence. We observe, in particular, that neopeptides associated to highly recurrent resistance mutations are predicted more likely to be HLA-presented across the general population than neopeptides generated by highly recurrent driver mutations (see also **Supplementary Figures 3, 4** for different levels of recurrence in these two sets of mutations). In **Supplementary Figures 5** and **6**, respectively, we show the PMHBR score distributions for the datasets in **Figure 1** when using only the HLA-A and HLA-B allotypes and when using all HLA haplotypes from patients taken from the TCGA dataset (46), respectively (**Supplementary Table 6** for the PMHBR scores). In **Supplementary Figures 7, 8**, instead, we calculate the percentage of 1000G healthy individuals that are predicted (IBR score < 0.5, *Methods*) to HLA-present at least one of the neopeptides associated to a given mutation and plot the corresponding distributions for each of the five datasets. In all instances, we observe similar trends as those seen in **Figure 1**. Only when excluding from the resistance set those mutations that occur in the BCR-ABL1 fusion gene (constituting about half of the whole) differences with respect to the other sets of mutations appear to be less significant suggesting that BCR-ABL1 mutations are on average particularly likely to be HLA-presented (**Supplementary Figure 9**). At the same time, when plotting PMHBR scores for each resistance gene separately, we see that additional genes contribute low PMHBR scores to the resistance mutation dataset (e.g., ALK, MET, SMO etc.) (**Supplementary Figure 10**).

**Figure 2:**
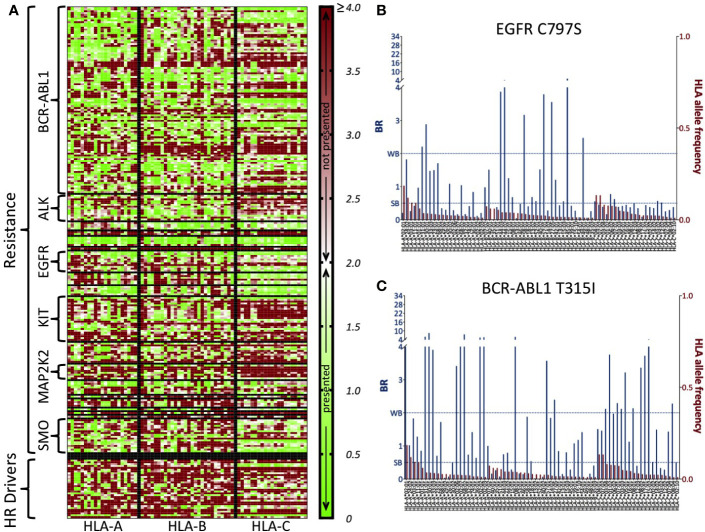
BR score-based HLA-presentation profiles of all COSMIC resistance mutations and highly recurrent (HR) driver mutations. **(A)** Heatmap of BR scores for 226 resistance mutations and 32 HR driver mutations (rows) versus 70 common HLA allotypes (those with frequency >1% in 1000G, columns). Low BR values (green) indicate high predicted likelihood of HLA-presentation of at least one neopeptide associated to the mutation. High BR values (red) indicate that there is no neopeptide associated to the mutation that is predicted likely to be HLA-presented. The middle point in the scale (white) corresponds to BR = 2.0, which is the threshold for peptide presentation (<2.0 means presentation, >=2.0 means no presentation). Values of BR above 4 are all colored with the same shade of red as BR = 4.0. HLA allotypes are ordered according to decreasing frequency in 1000G and separately for the three HLA-A, -B, and -C genes (vertical lines). Resistance mutations are grouped according to the gene they occur in (horizontal lines). For the sake of readability, we write only some of the resistance gene names and HR driver mutations are grouped all together. **(B)** BR scores for resistance mutation C797S in EGFR and **(C)** T315I in BCR-ABL1. Blue bars (primary y-axis) represent the BR scores of the mutation with respect to the HLA allotypes reported on the x-axis. Red bars (secondary y-axis) represent the frequency of each HLA allotype in the 1000G dataset. We report BR scores for only the HLA-A, -B, and -C allotypes that have frequency >1% in 1000G. The two dotted lines mark elution rank value limits for strong likelihood of presentation (SB, i.e., BR score < 0.5) and weaker likelihood of presentation (WB, i.e., 0.5<= BR score < 2.0). Note that in panels **(B, C)**, for the sake of readability, the part of the primary y-axis corresponding to values of BR above 4 is compressed.

In **Figure 2A**, we show a heatmap with the BR score-based HLA-presentation profiles (*Methods*) of all 226 COSMIC resistance mutations along with the 32 HR driver mutations for 70 common HLA allotypes. It can be seen how neopeptides from HR driver mutations are quite often predicted not to be HLA-presented (red), and also that differences exist in HLA-presentation profiles of resistance mutations in different genes (compare with **Supplementary Figure 10**). In **Figures 2B, C** as examples, we show the BR score-based HLA-presentation profile of two resistance mutations of particular clinical relevance. The EGFR C797S mutation represents a major challenge for treatment of osimertinib-resistant tumors in non-small cell lung cancer (48). T315I, until approval of the third-generation inhibitor ponatinib (59), was the most common mutation associated with resistance to BCR-ABL1 inhibitors (60, 61) and is the second most commonly reported mutation in the COSMIC dataset. From these profiles, we see that both mutations are predicted to generate neopeptides that produce low BR scores (that is, are predicted to have high presentation likelihood) across most HLA allotypes that are being considered. For comparison, we show the BR score-based HLA-presentation profiles of two common driver mutations, chosen as the most commonly observed in the TCGA dataset, in **Supplementary Figure 11**.

**Figure 3:**
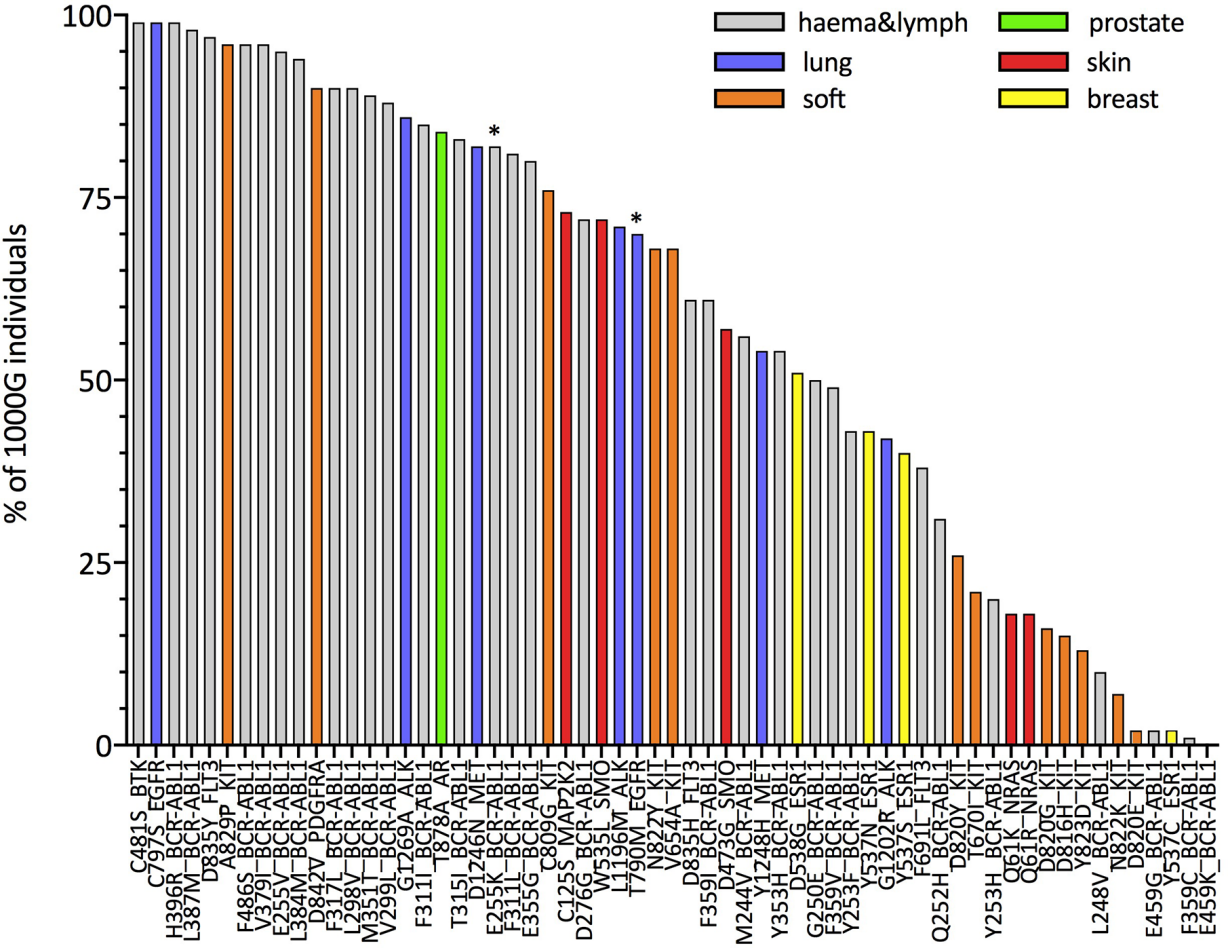
Estimates for the percentage of individuals in the general population predicted to HLA-present resistance mutation-associated neopeptides. For each mutation, the histogram illustrates the percentage of individuals in the 1000G dataset with an IBR < 0.5 (the IBR score is defined in *Methods*). Mutations on the x-axis are ordered according to decreasing percentages of individuals. We plot only mutations that have been observed in at least five patients (according to COSMIC). Colors indicate the different tumor tissues in which the resistance mutations have been observed; “haema&lymph” stands for hematopoietic and lymphoid tissue. Asterisks (*) mark mutations that have been shown to elicit T-cell responses in previous works (32–34).

**Figure 4:**
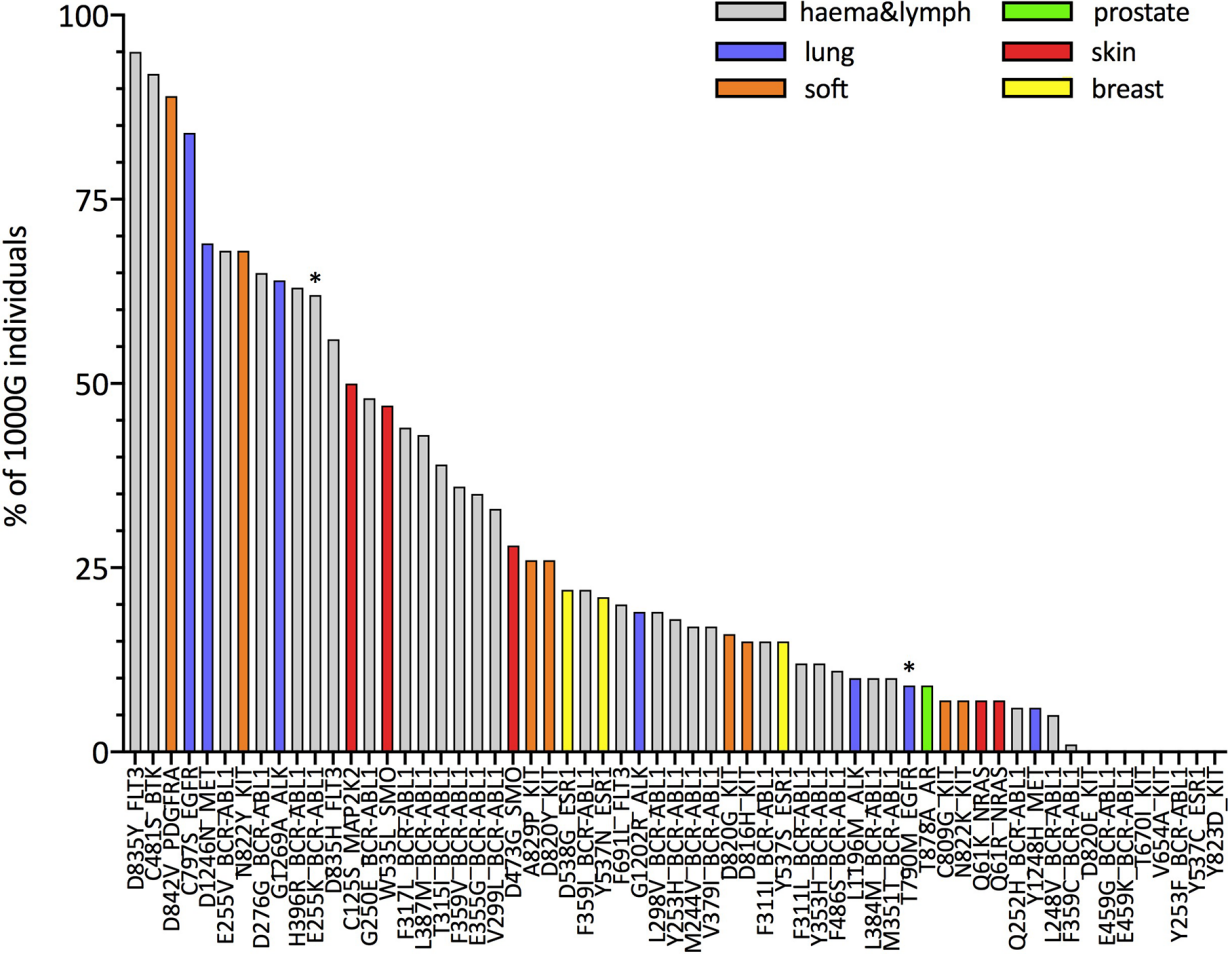
Population-wide comparison of HLA class I presentation likelihood between resistance mutation-associated neopeptides and their corresponding wild type peptides. For each mutation, the histogram illustrates the estimated percentage of individuals for which at least one mutant peptide-wild type peptide pair exists such that the MinRank of the mutant peptide is <0.5 and the MinRank of the corresponding wild type peptide is ≥0.5 (see *Methods* for definitions). Mutations on the x-axis are ordered according to decreasing percentages of individuals. We plot only mutations that have been observed in at least five patients (according to COSMIC). Colors indicate the different tumor tissues in which the resistance mutations have been observed; “haema&lymph” stands for hematopoietic and lymphoid tissue. Asterisks (*) mark mutations that have been shown to elicit T-cell responses in previous works (32–34).

Next, we focus on resistance mutations with at least five occurrences in COSMIC as those that would be of particular interest in the context of off-the-shelf targeted immunotherapeutic approaches. We show, this time separately for each of these resistance mutations, the percentage of 1000G healthy individuals predicted to present the associated neopeptides (IBR score < 0.5, 61 mutations in total; **Figure 3** and percentages for all 226 mutations in **Supplementary Table 7**). The same analysis when considering only HLA-A and HLA-B allotypes for calculating the IBR score can be found in **Supplementary Figure 12**. We first notice that our results are in line with previous studies that showed that several BCR-ABL1 mutations (32) [and T790M in EGFR (33, 34)] are likely to be HLA-presented (**Supplementary Figure 13**), although those studies considered a much smaller set of HLA allotypes. Second and more importantly, we show that several other resistance mutations occurring in different tumor tissues (**Figure 3**) and in several other genes (**Supplementary Figure 14**) are also predicted as likely to be HLA-presented across the population. In general, we can see that 39 of 61 mutations in **Figure 3** generate neopeptides that are predicted to be HLA-presented by at least 50% of individuals in our 1000G dataset. These 39 mutations occur in six different tumor tissues and across 12 different genes. When considering a more relaxed threshold for HLA-presentation (IBR < 2.0) all but one of these mutations are predicted to be presented by at least half of individuals in the 1000G dataset (**Supplementary Figure 15**). As an example, neopeptides associated with the osimertinib resistance mutation C797S in EGFR and to T315I in BCR-ABL1 are predicted to be presented in 99 and 83% of individuals, respectively (for an IBR threshold <0.5). Interestingly, the two mutations that have been previously validated as able to elicit T-cell responses in healthy donors and patients alike (E255K in BCR-ABL1 and T790M in EGFR) are not among our top-ranked ones (21^st^ and 29^th^, respectively). We next investigate the possibility that resistance mutation neopeptides, while likely to be HLA-presented in the general population, may be subjected to tolerance, in which case they would not be immunogenic. Under normal circumstances, tolerance ensures that there are no T-cells that can recognize germline wild type peptides, thus preventing auto-immune responses (62). Since neopeptides are generated by somatic mutations, they are very likely to differ from any germline wild type peptide. At the same time, neopeptides originating from missense mutations, such as those that we analyze here, differ from wild type peptides only by a single amino acid substitution. Given that T-cell binding properties allow for at least some promiscuity in peptide binding affinity (63), missense mutation-associated neopeptides might still be subjected to some degree of tolerance. A common way to identify neopeptides that are more likely to be immunogenic is to select for those that have wild type counterparts with low HLA-presentation likelihood (64). The rationale is that if a wild type peptide is poorly presented, T-cells that bind to it and hence possibly to very similar peptides are less likely to have been negatively selected. It is important to stress, however, that even a neopeptide for which the wild type counterpart is strongly HLA-presented may be immunogenic if the mutation it carries makes it eligible to binding by a different T-cell pool with respect to the wild type. In **Figure 4**, we compare the presentation likelihood of resistance mutation-associated neopeptides with that of their wild type counterparts across 1000G healthy individuals (only for the 61 mutations recorded in at least five patients in COSMIC; values for all mutations are in **Supplementary Table 7**). In particular, we report the percentage of individuals in which at least one mutant peptide is predicted highly likely to be presented (MinRank < 0.5) while the corresponding wild type peptide is not highly likely to be presented (MinRank ≥ 0.5). We can see that for 13 mutations the percentage of individuals is at least 50% (see **Supplementary Figure 16** for HLA-A and HLA-B only). Again, mutations previously shown to be immunogenic do not exhibit the highest rankings in this plot (BCR-ABL1 E255K is 11^th^ and EGFR T790M is 44^th^). In **Supplementary Figures 17–20**, we show the same analysis when using alternative criteria for evaluating the difference between mutant and wild type peptides. Finally, in **Supplementary Figures 21, 22**, we show the resistance mutations’ associated neopeptides (length 8 to 11) that we predict to have the highest percentage of individuals more likely to present them (MinRank < 0.5 or MinRank < 2.0) than to present their wild type counterparts (MinRank ≥ 0.5 or MinRank ≥ 2.0, respectively). With respect to the previously validated neopeptides associated with the E255K BCR-ABL1 and T790M EGFR resistance mutations we observe the following. We predict the BCR-ABL1-associated neopeptide KVYEGVWKK to be highly likely to be HLA-presented by the HLA-A*03:01 allotype, or the allotype for which immunogenicity has been validated (%rank = 0.005), and almost 100-fold more likely to be presented than its wild type counterpart EVYEGVWKK (%rank = 0.33). However, since the wild type peptide is also predicted as likely to be presented (%rank < 0.5), KVYEGVWKK does not fare high in our plots that use a fixed threshold for both mutant and wild type peptides while it scores definitely better when considering a <0.5% rank threshold for the mutant and simply asking that the wild type has higher ranking than the mutant peptide (**Supplementary Figure 23**). In contrast, we don’t predict the T790M-associated mutant peptides that have been previously validated as immunogenic (MQLMPFGCLL, LIMQLMPFGCL, IMQLMPFGC) to be likely to be presented by the experimentally validated HLA-A*02:01 allotype (%ranks = 2.12, 16.0, and 5.74, respectively). We do however observe a separate, not previously tested T790M-associated neopeptide (LTSTVQLIM) as one that has high immunogenic potential across the population (third from top in **Supplementary Figure 22**).

**Figure 5:**
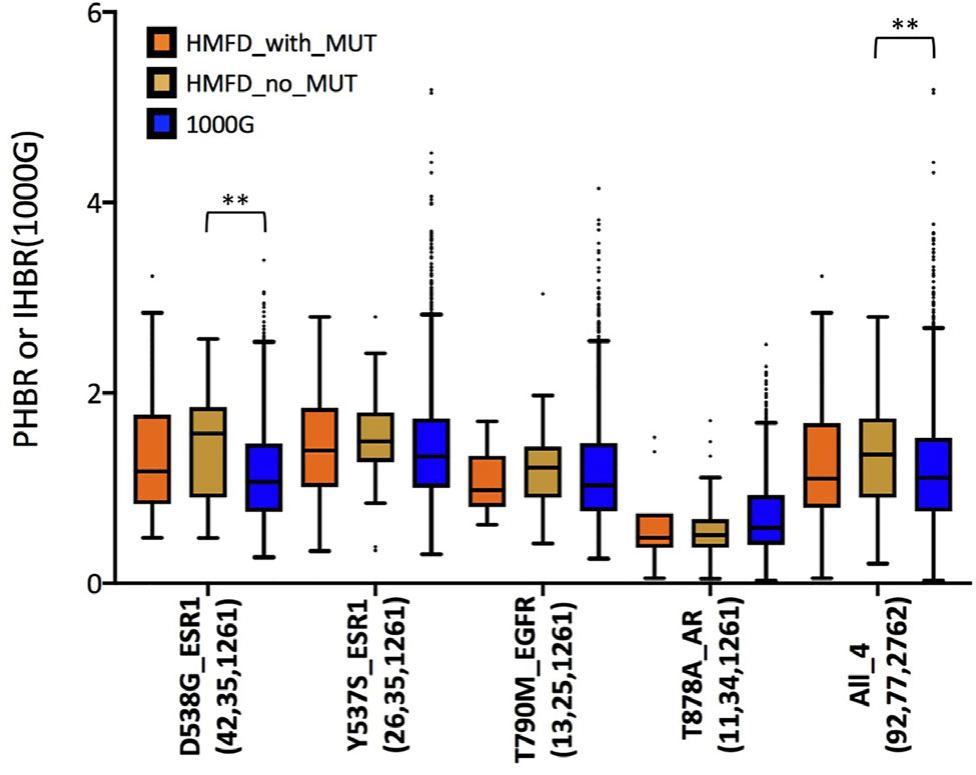
Distribution of PHBR scores in different groups of the Hartwig Medical Foundation Database (HMFD) patients treated with targeted drugs. Lower PHBR values correspond to a higher likelihood of being presented by HLA class I complexes. For each mutation, “HMFD_with_MUT” and “HMFD_no_MUT” represent patients treated with similar targeted drugs, however, “HMFD_with_MUT” (orange) are patients that developed the mutation while “HMFD_no_MUT” (yellow) are patients that did not develop it. “1000G” (blue) are all the healthy individuals in our 1000G dataset. On the x-axis, for each mutation we report in parentheses the number of patients/individuals considered in each of the three groups (the order is “HMFD_with_MUT", "HMFD_no_MUT", "1000G"). Note that when considering all four mutations together, for a fair comparison, “no_MUT” patients and “1000G” healthy individuals have been sampled (randomly with replacement) so to obtain a proportion of scores derived from each mutation similar to the one observed among “with_MUT” patients. “HMFD_no_MUT” for ESR1 D538G and Y537S are the same group of breast cancer patients. Asterisks indicate significance of pairwise differences between score distributions calculated using Mann-Whitney tests with no multiple comparison adjustment. (**) stands for p-value < 0.01. Pairwise tests were performed only between distributions in the same mutation group. The lower and higher edges of each Tukey box represent the 25 and 75% percentile value, respectively. The horizontal line inside each box represents the median value.

**Figure 6:**
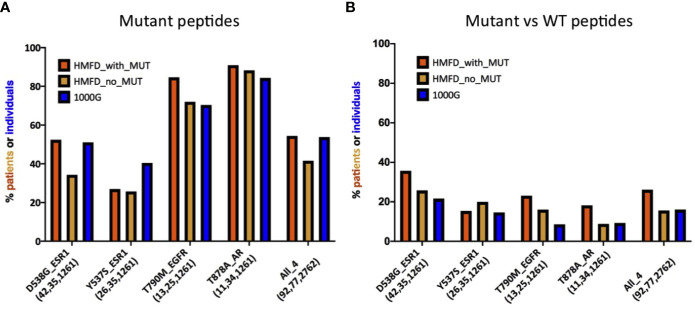
**(A)** Estimates for the percentage of patients or individuals predicted to HLA-present neopeptides associated to four different resistance mutations. Groups of patients and of healthy individuals as well as notations are as described in **Figure 5**. For each mutation in each group, the histogram illustrates the percentage of patients with a PBR < 0.5 [HMFD patients] or IBR < 0.5 (1000G). Both IBR and PBR scores are defined in *Methods*. **(B)** Comparison between mutant peptides and corresponding wild type peptides for the same mutations and groups of patients/individuals shown in panel **(A)**. For each mutation and each group, the histogram illustrates the estimated percentage of patients or healthy individuals for which at least one mutant-wild type peptide pair exists such that the MinRank of the mutant peptide is <0.5 and the MinRank of the corresponding wild type peptide is ≥0.5 (see *Methods* for definitions). Estimates in **(A, B)** for 1000G are the same as those reported for these mutations in **Figures 3** and **4**, respectively.

So far, we have shown that several recurrent resistance mutations are predicted to have immunogenic potential in a significant fraction of healthy individuals in the general population (i.e., 1000G). An important question, however, is whether this is true also in patients that carry the mutations or, rather, resistance mutations are observed primarily in patients with HLA haplotypes unlikely to present the associated neopeptides. Although evidence of negative selection has been reported for driver mutations (45), it would seem less probable for resistance mutations which typically appear later during cancer evolution, when immune-evasion by the tumor is likely to have already occurred. This is in line with our observation that, contrary to what seen for driver mutations, the PMHBR scores of resistance mutations do not increase with increasing levels of recurrence (**Figure 1** and **Supplementary Figures 1, 3, 4**). We obtain a similar result when considering a subset of mutations that occur within the same gene and in response to the same drug, that is, when removing the confounding effect of different dates of approval for different drugs on the number of patients in which a mutation was observed (see the plot in **Supplementary Figure 24** where we compare PMHBR scores of all BCR-ABL1 mutations that confer resistance to the drug imatinib, differences are not significant, Kruskal-Wallis and Dunn’s). Even so, we decided to investigate this important aspect more directly, by gaining access to the Hartwig Medical Foundation database (HMFD) (44). The HMFD is a large cancer resource that includes patients who have undergone targeted therapy and for which the HLA haplotypes of the patients can be predicted from WGS germline data. We selected four recurrent resistance mutations in three different solid tumor tissues (*Methods*): i) ESR1 D538G (breast), ii) ESR1 Y537S (breast), iii) EGFR T790M (lung), and iv) AR T878A (prostate). We extracted data for respectively i) 42, ii) 26, iii), 13 and iv) 11 patients carrying these mutations and we predicted their HLA haplotypes (*Methods*). As controls, we additionally considered a set of HMFD patients with breast, lung, or prostate cancer treated with the same (or similar) targeted therapy as the groups above but that did not develop the corresponding resistance mutations. In **Figure 5**, we compare the patient-specific PHBR score distribution (*Methods*) for patients with and without the mutations for the four mutations separately and, additionally, for all mutations together. Further, for all of the above cases, we report the PHBR distributions calculated on healthy 1000G individuals (**Supplementary Figure 25** for violin plots; PHBR scores are reported in **Supplementary Table 5**). We observe that none of the pairwise differences in PHBR scores between HMFD patients with and without the mutations are significant (Mann-Whitney tests), suggesting that these resistance mutations don’t tend to occur preferentially in patients that have lower likelihood of presenting them. Some differences with 1000G-based estimates are to be expected as a result of the different population structure in HMFD *versus* 1000G, the former collecting data from patients in the Netherlands and the latter covering individuals from a wide range of world populations. Despite this, when comparing the PHBR scores of the HMFD patients’ groups with those of 1000G individuals only pairwise differences between HMFD_no_MUT and 1000G in the D538G_ESR1 and All_4 sets are significant (both with p-value < 0.01, Mann-Whitney tests with no multiple comparison adjustment). In general, we see how across mutations PHBR scores appear to scale similarly to what estimated from 1000G healthy individuals, with ESR1 mutations generally less likely to be HLA-presented than T790M EGFR and T790M EGFR less likely to be HLA-presented than T878A AR (note that the higher the PHBR score, the lower the likelihood to be presented; compare also with **Figure 3**). PHBR score distributions of drivers and passengers in HMFD patients (*Methods*) show trends that are similar to the ones seen for PMHBR scores calculated on 1000G individuals in **Figure 1** (**Supplementary Figures 26A, B** where, merely for completeness, we also report PHBR scores of the four resistance mutations we have analyzed). In **Figure 6A**, we compare the percentage of HMFD patients with and without the four resistance mutations that are predicted to HLA-present (PBR < 0.5) at least one of the associated neopeptides along with the corresponding percentages in 1000G healthy individuals. As expected, these estimates reflect the PHBR score distributions of **Figure 5** (**Supplementary Figure 27A** for similar plots considering only HLA-A and HLA-B). In **Figure 6B**, we compare the percentage of patients that are predicted to HLA-present (at least) one of the neopeptides associated to the mutation (MinRank < 0.5) and not highly likely to present the corresponding wild type peptide (MinRank ≥ 0.5) (**Supplementary Figure 27B** for HLA-A and HLA-B only). In this case, patients carrying the resistance mutations are predicted to have slightly higher likelihood of presentation than both patients without the mutations and 1000G healthy individuals although this could to some extent be due to statistical fluctuations. Next, we look for evidence of selection within individual patients rather than within individual mutations. To this end, we take advantage of the fact that two resistance mutations in our HMFD set, D538G and Y537S in ESR1, occur in response to the same targeted drug-therapy. We ask whether in patients that carry only one of the two mutations, the one that is observed has on average a higher PHBR score than the one that is not observed. If so, this would suggest that, in a patient, these ESR1 mutations are selected to have a lower likelihood of presentation. Again, we find no significant difference (Wilcoxon tests) between the PHBR scores of mutation pairs (both when using all data, **Supplementary Figures 28A, B**, and when using a 50%-50% balanced dataset, not shown; see **Supplementary Table 5** for all the raw data). Overall, in the four recurrent resistance mutations we have analyzed in HMFD patients we appear to find no evidence of negative immune selection. Also, comparison with 1000G healthy individuals suggests that analysis performed on the latter can be informative of the immunogenic potential in cancer patients for mutations in the wider COSMIC dataset.

The complete list of resistance mutations that we analyze here along with estimates of the percentage of individuals in the 1000G dataset that are likely to present their associated neopeptides and, separately, that are more likely to present their associated neopeptides than their wild type counterparts can be found in **Supplementary Table 7**. Lists of resistance mutation-associated neopeptides that are more likely to be HLA-presented than their wild type counterparts are instead reported in **Supplementary Table 8** (peptides associated to resistance mutations observed in at least five patients in COSMIC and estimated to be presented by at least 1% of 1000G individuals).

## Discussion and Conclusions

Cancer immunotherapies seek to invigorate a patient’s immune response against the tumor (5). This response is typically mediated by tumor antigens that originate from the cancer cells’ aberrant immunopeptidome. Cancer drug resistance mutations are one class of somatic variants that generate tumor-specific, potentially immunogenic antigens. Previous studies showed that two resistance mutations, E255K in BCR-ABL1 and T790M in EGFR, are indeed immunogenic (32–34); additionally, Cai et al. (32) suggested that this property might be shared by a larger number of BCR-ABL1 resistance mutations. These previous studies looked at presentation by a small number of class I HLA allotypes [five HLA-A and three HLA-B allotypes in (32) and only HLA-A*02:01 in (33, 34)]. We asked whether these immunogenic properties could be shared by a larger number of cancer drug resistance mutations and when considering a much larger set of class I HLA allotypes. Using *in silico* predictions we present, for the first time, a general survey of the immunogenic potential of 226 missense resistance mutations associated with several genes (19 total) and tissues (9 total). We show that many of these mutations generate neopeptides that are predicted to be HLA-presented by a large proportion of the general population. Additionally, for several resistance mutations and in a significant percentage of individuals, these potential neoantigens are predicted more likely to be HLA-presented than their wild type counterparts, and are therefore less likely to fall under central or peripheral tolerance. Importantly, by looking at data from the Hartwig Medical Foundation database, we found no evidence that resistance mutations undergo negative selection by the immune system in resistant patients. This is in agreement with the observation that HLA-presentation scores of resistance mutations in the general population and their level of recurrence in patients do not appear to be correlated.

In this work, we have considered only missense mutations, which constitute the vast majority of drug resistance mutations currently annotated in COSMIC; however, insertions and deletions are also known to confer resistance to some drugs. In a recent publication, for example, we showed that revertant frameshift mutations in patients treated with PARP-inhibitors could encode neopeptides predicted to be HLA-presented in the general population (65). Notably, neopeptides generated by this type of somatic alterations, if presented, would be more likely to be immunogenic as they will generally differ substantially from any wild type protein peptide (66).

Our study comes with a number of limitations. The most obvious one is that our results are based on computational predictions (64). Although the most recent breed of prediction methods (such as the NetMHCpan-4.0 program that we use here) integrate peptides’ HLA-elution mass spectrometry data, they are still likely to over-predict the number of presented peptides (67–69). Also, higher presentation likelihood with respect to the corresponding wild type peptide is probably a poor proxy for a mutant peptide’s immunogenicity (i.e., actual recognition by T-cells). Despite these important *caveats*, as mentioned above, computational predictions have been used previously to identify potentially immunogenic neopeptides from the BCR-ABL1 E255K and EGFR T790M resistance mutations, which were later proved effective in priming naïve T-cells (32–34); two of these studies showed, additionally, that T-cells specific for resistance neoantigens could be detected in patients (32, 33). Further, methods that predict HLA-presentation have been widely adopted in and instrumental to studies that showed neoantigen load correlation with CBT response (70), established the occurrence of immune-evasion by neoantigen elimination (71–73), investigated personalized cancer vaccines against melanoma and glioblastoma in small clinical trials (21–24) and suggested negative immune-selection for highly recurrent driver mutations (45). In particular, several studies have shown that lists of predicted neoantigens are indeed enriched in neopeptides capable of stimulating T-cell responses both *in vitro* and *in vivo* (20–24, 74). Another potential concern is the fact that some of the proteins in which resistance mutations to current targeted therapies occur are membrane-inserted. Membrane proteins are generally believed to undergo degradation in lysosomes (75) rather than *via* the ubiquitin-proteasome pathway, which leads to HLA class I presentation of protein peptides. There is compelling evidence that nevertheless membrane protein peptides are presented by HLA class I complexes (76). Furthermore, tumors are known to develop immune-escape strategies including, but not limited to, loss of heterozygosity at the HLA locus, down-regulation of HLA genes and up-regulation of immune checkpoints (3). As a consequence, we cannot exclude that patients that develop mutations conferring resistance to targeted therapies carry tumors that may be refractory to a targeted immunotherapeutic approach [see, for example, results in (33)]. In recent years, however, treatments such as CBTs (6) have become available that are able of thwarting one of tumors’ immune escape mechanisms by restoring immune surveillance in at least some groups of patients and could thus potentially be used in combination with more targeted immunotherapies. Finally, there are limitations in terms of the composition of the mutation datasets that we have analyzed in particular for what concerns representation of mutations resistant to different drugs and occurring in different tumor types (for example, COSMIC does not include all known resistance mutations and the frequency of occurrence for mutations resistant to more recently approved drugs is likely to be grossly underestimated).

In conclusion, expanding on previous studies, we have presented data that suggests that resistance mutation-associated neoantigens could be particularly interesting targets for precision immunotherapies such as cancer vaccines (77). Most recent work in the field has focused on tumor neoantigens associated with protein-modifying passenger mutations (21–24). However, vaccines derived from passenger mutations, which are private, would represent fully personalized treatments with potentially high development costs and scale-up issues for translation into the clinic (43). In contrast, recurrent neoantigens such as those potentially derived from resistance mutations could serve as a basis for developing off-the-shelf vaccines, which could be used in combination with targeted drug therapies, as well as with other types of immunotherapies. We believe that the recent advances in cancer immunotherapy and the ever-increasing number of approved targeted therapies provide an unprecedented background on which to test the potential immunogenicity of recurrent resistance mutations.

## Data Availability Statement

All datasets generated for this study are included in the article/**Supplementary Material**.

## Author Contributions

All authors listed have made a substantial, direct, and intellectual contribution to the work and approved it for publication.

## Funding

MP and SL are funded by the Wellcome Trust (105104/Z/14/Z). AM and VJ are supported by Cancer Research UK (C16708/A21855). MP and VJ are supported for this project by a Schottlander Innovation Award from The Schottlander Research Charitable Trust.

## Conflict of Interest

The authors declare that the research was conducted in the absence of any commercial or financial relationships that could be construed as a potential conflict of interest.
