# Supplementary material for: The Immunogenic Potential of Recurrent Cancer Drug Resistance Mutations: An In Silico Study
Source: Front Immunol. 2020 Oct 8;11:524968. doi: 10.3389/fimmu.2020.524968 (PMC7578429; doi:10.3389/fimmu.2020.524968)
Supplement: Supplementary file 1 [file DataSheet_1.pdf]

Supplementary Material

Supplementary Figures

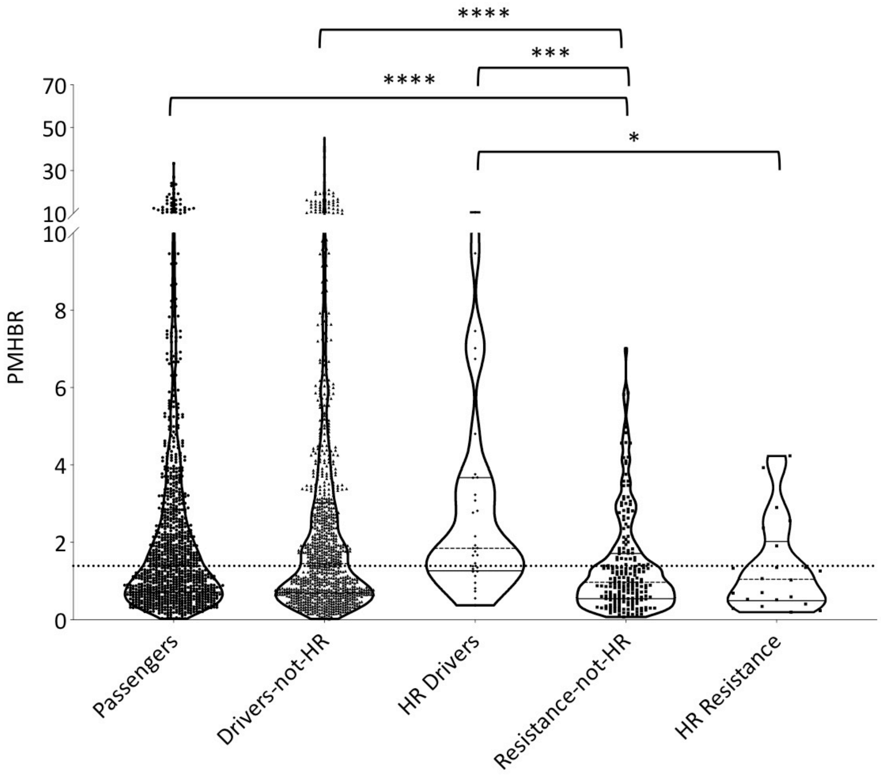

Supplementary Figure 1. *Same as Figure 1 but using violin plots and staggered dots.* Note that, for the sake of readability, the part of the y-axis corresponding to values of PMHBR above 10 is compressed. The dotted horizontal line is a guide for the eye and corresponds to the value of the median of the distribution for passenger mutations. Asterisks indicate significance of differences between PMHBR score distributions calculated using a Kruskal-Wallis test followed by Dunn's *post hoc* test. p-values are adjusted for multiple testing (all vs all). (*) stands for p-value <0.05, (***) for p-value<0.001 and (****) for p-value<0.0001. Horizontal lines within each violin plot indicate 25th percentile, median and 75th percentile (bottom, middle and top, respectively).

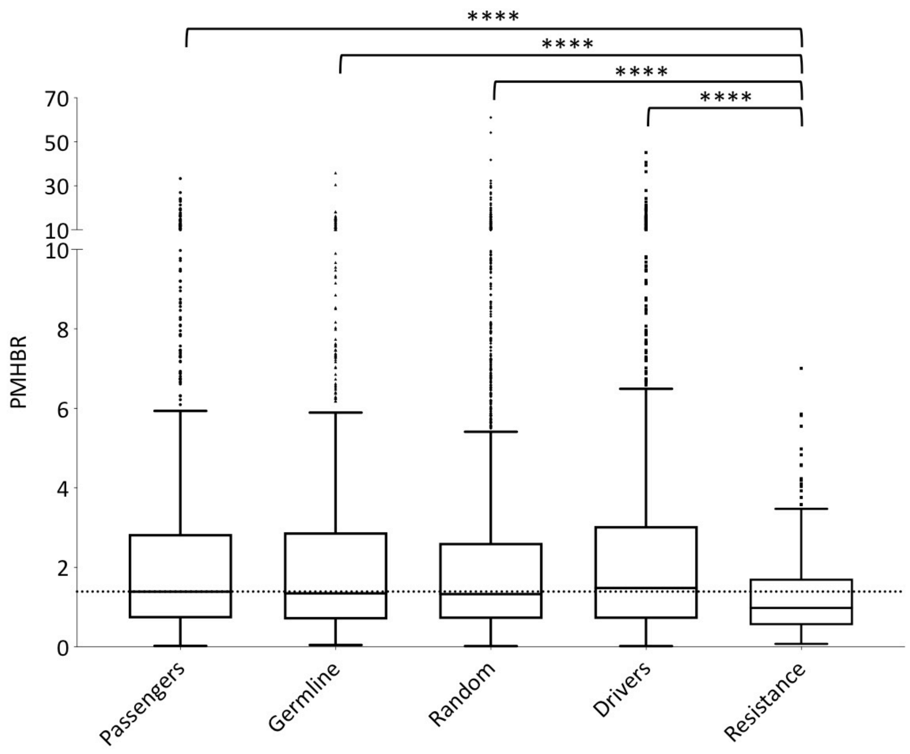

Supplementary Figure 2. *Similar to Figure 1 when adding PMHBR distributions for germline SNPs and random mutations and plotting the PMHBR for full sets of driver and resistance mutations.* Note that, for the sake of readability, the part of the y-axis corresponding to values of PMHBR above 10 is compressed. The dotted horizontal line is a guide for the eye and corresponds to the value of the median of the distribution for passenger mutations. Asterisks indicate significance of differences between PMHBR score distributions calculated using a Kruskal-Wallis test followed by Dunn's *post hoc* test. (****) stands for p-value<0.0001

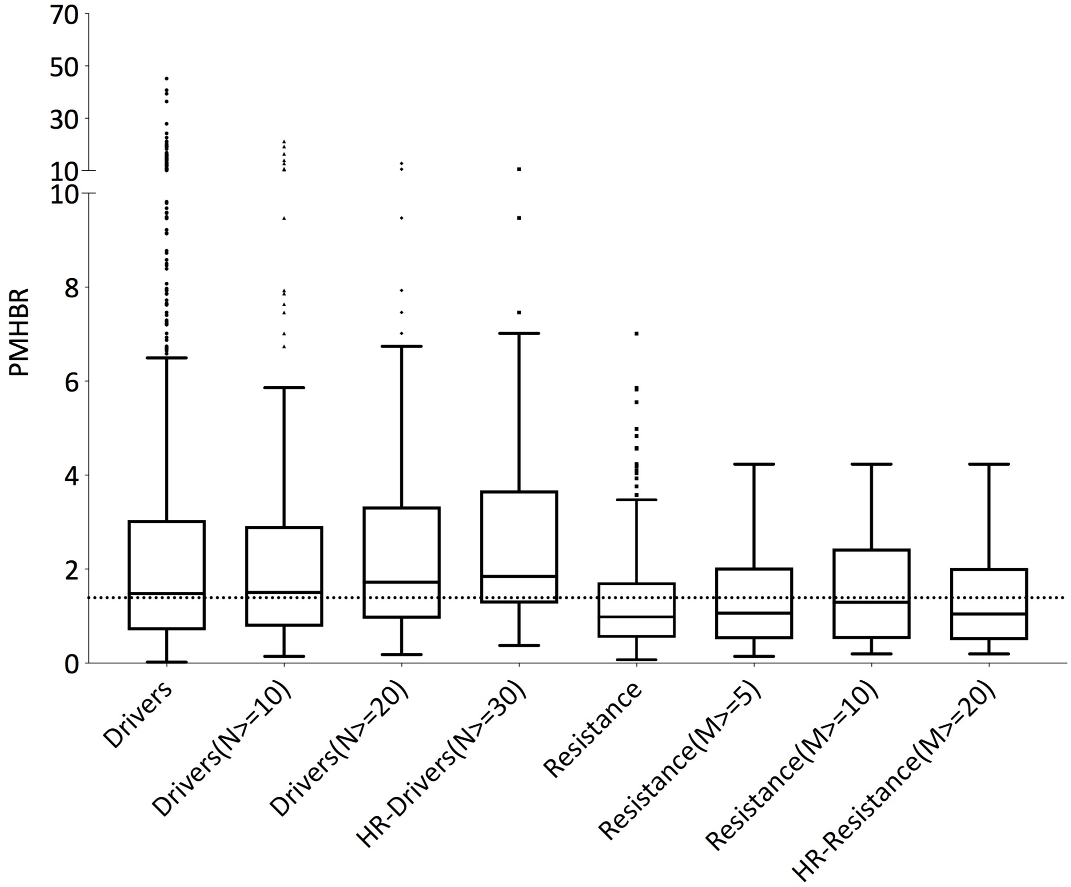

Supplementary Figure 3. *Similar to Figure 1 but plotting driver and resistance mutations sets of increasing levels of recurrence. N* stands for number of TCGA patients in which a putative driver mutation (**Methods**) has been observed and *M* for the number of times a resistance mutation has been recorded in COSMIC. For both driver and resistance mutations each set with increasing N and M, respectively, is a subset of the previous one. Note that *N* and *M* are only roughly comparable because of differences in size and composition between the underlying datasets. Additionally, the number of records *M* in COSMIC for a given resistance mutations is affected by the date of approval of the drug the mutation is resistant to (the more recent is the date, the more likely is for *M* to be low). Note that, for the sake of readability, the part of the y-axis corresponding to values of PMHBR above 10 is compressed. The dotted horizontal line is a guide for the eye and corresponds to the value of the median of the distribution for passenger mutations as seen in Figure 1.

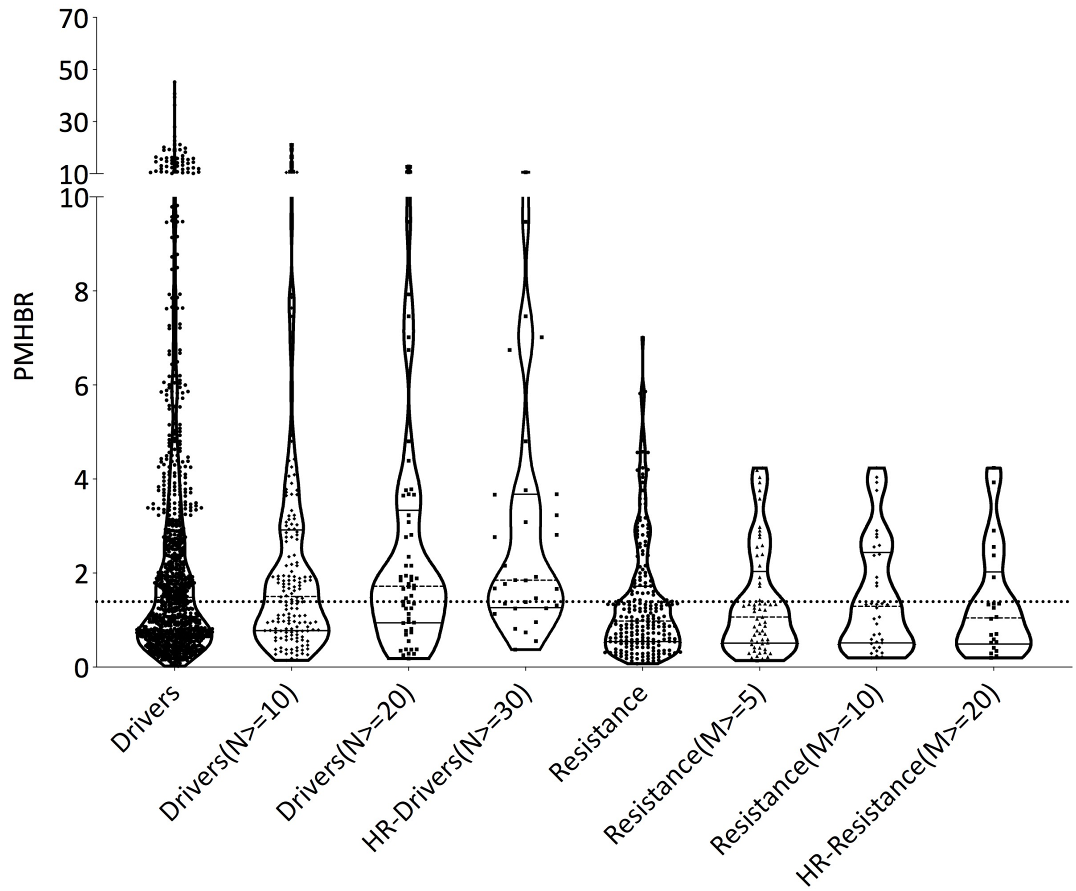

Supplementary Figure 4. *Same as Supplementary Figure 3 but using violin plots and staggered dots.* Note that, for the sake of readability, the part of the y-axis corresponding to values of PMHBR above 10 is compressed. The dotted horizontal line is a guide for the eye and corresponds to the value of the median of the distribution for passenger mutations as seen in Figure 1.

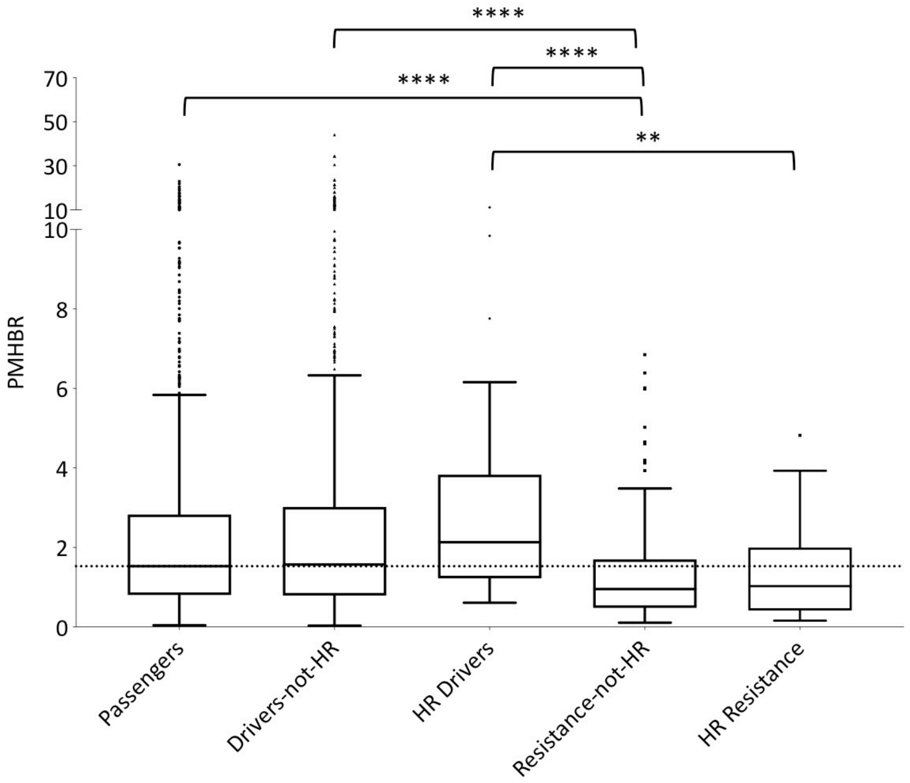

Supplementary Figure 5. *Equivalent to Figure 1 when using only HLA-A and HLA-B for calculating the PMHBR scores (Equation 1).* Note that, for the sake of readability, the part of the y-axis corresponding to values of PMHBR above 10 is compressed. The dotted horizontal line is a guide for the eye and corresponds to the value of the median of the distribution for passenger mutations. Asterisks indicate significance of differences between PMHBR score distributions calculated using a Kruskal-Wallis test followed by Dunn's *post hoc* test. p-values are adjusted for multiple testing (all vs all). (**) stands for p-value<0.01 and (****) for p-value <0.0001.

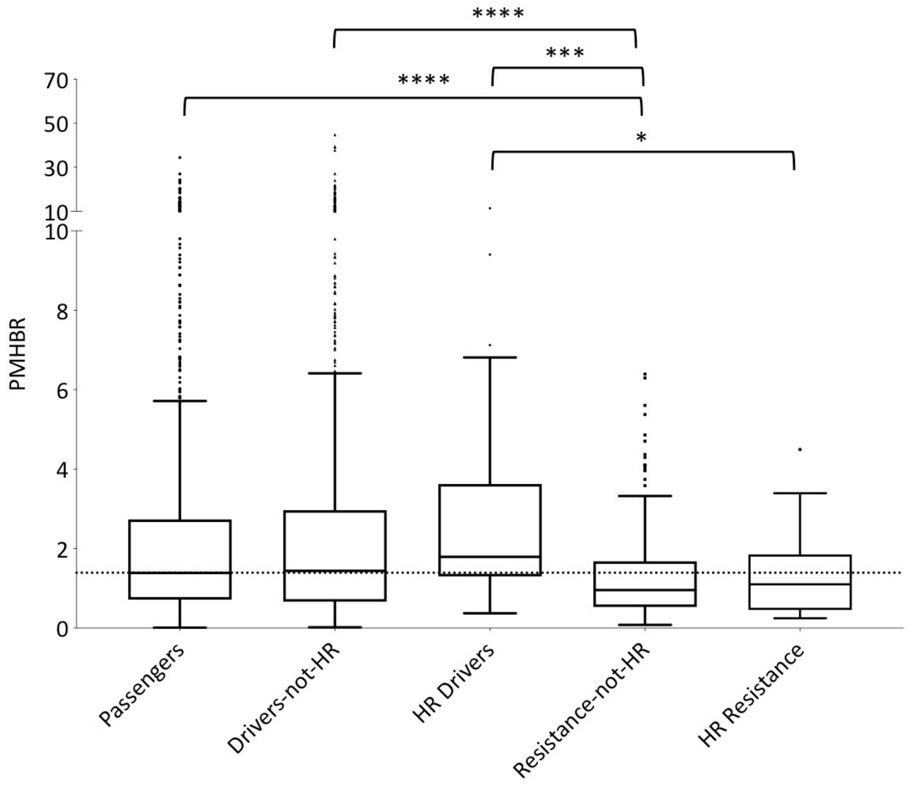

Supplementary Figure 6. *Equivalent to Figure 1 when using TCGA patients instead of 1000G individuals to calculate the PMHBR scores (Equation 1).* Note that, for the sake of readability, the part of the y-axis corresponding to values of PMHBR above 10 is compressed. The dotted horizontal line is a guide for the eye and corresponds to the value of the median of the distribution for passenger mutations. Asterisks indicate significance of differences between PMHBR score distributions calculated using a Kruskal-Wallis test followed by Dunn's *post hoc* test. p-values are adjusted for multiple testing (all vs all). (*) stands for p-value<0.05, (***)for p-value<0.001 and (****) stands for p-value<0.0001.

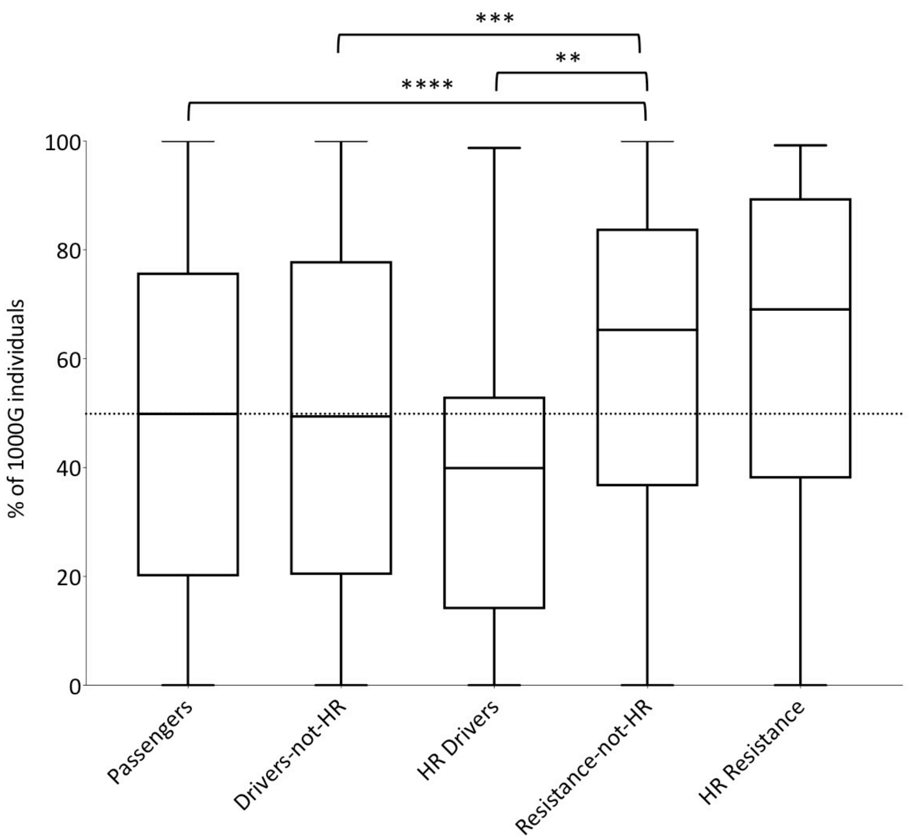

Supplementary Figure 7. *Comparison between the same mutation datasets of Figure 1 but calculating for each mutation the percentage of 1000G healthy individuals predicted to HLA-present at least one of its associated neopeptides (i.e. having an IBR<0.5,* ***Methods****)*. The dotted horizontal line is a guide for the eye and corresponds to the value of the median of the distribution for passenger mutations. Asterisks indicate significance of differences between PMHBR score distributions calculated using a Kruskal-Wallis test followed by Dunn's *post hoc* test. p-values are adjusted for multiple testing (all vs all). (**) stands for p-value<0.01, (***) for p-value<0.001 and (****) for p-value<0.0001.

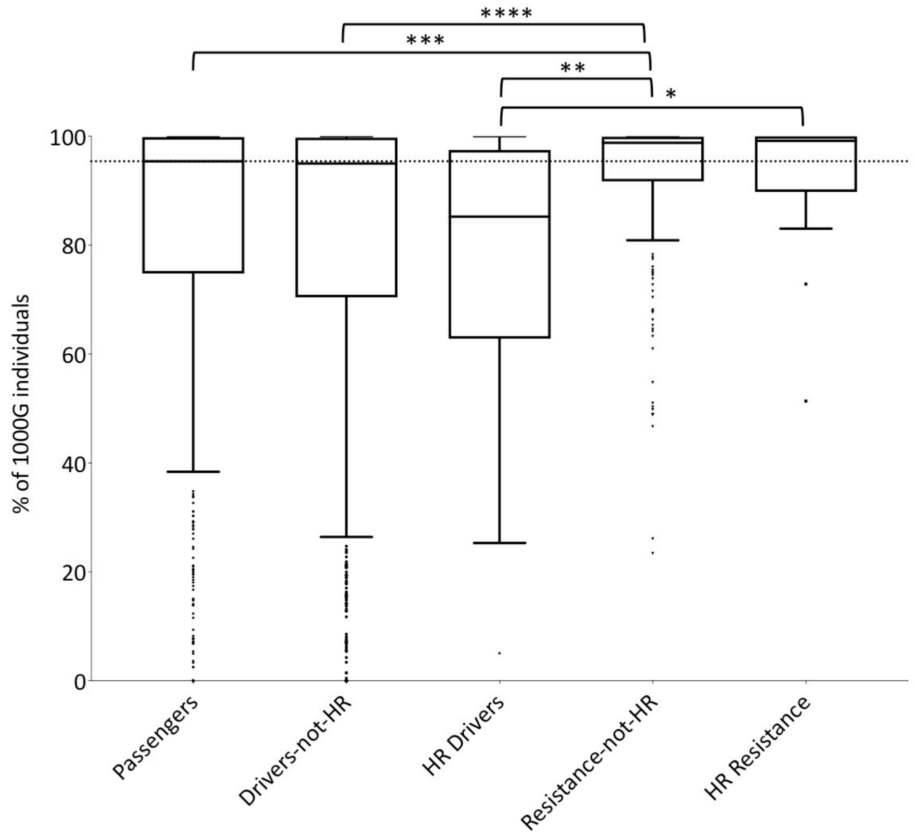

Supplementary Figure 8. *Equivalent to Supplementary Figure 7 when considering an IBR<2.0 as a criterion for HLA-presentation.* The dotted horizontal line is a guide for the eye and corresponds to the value of the median of the distribution for passenger mutations. Asterisks indicate significance of differences between PMHBR score distributions calculated using a Kruskal-Wallis test followed by Dunn's *post hoc* test. p-values are adjusted for multiple testing (all vs all). (*) stands for p-value<0.05, (**) for p-value <0..01, (***) for p-value<0.001 and (****) for p-value<0.0001.

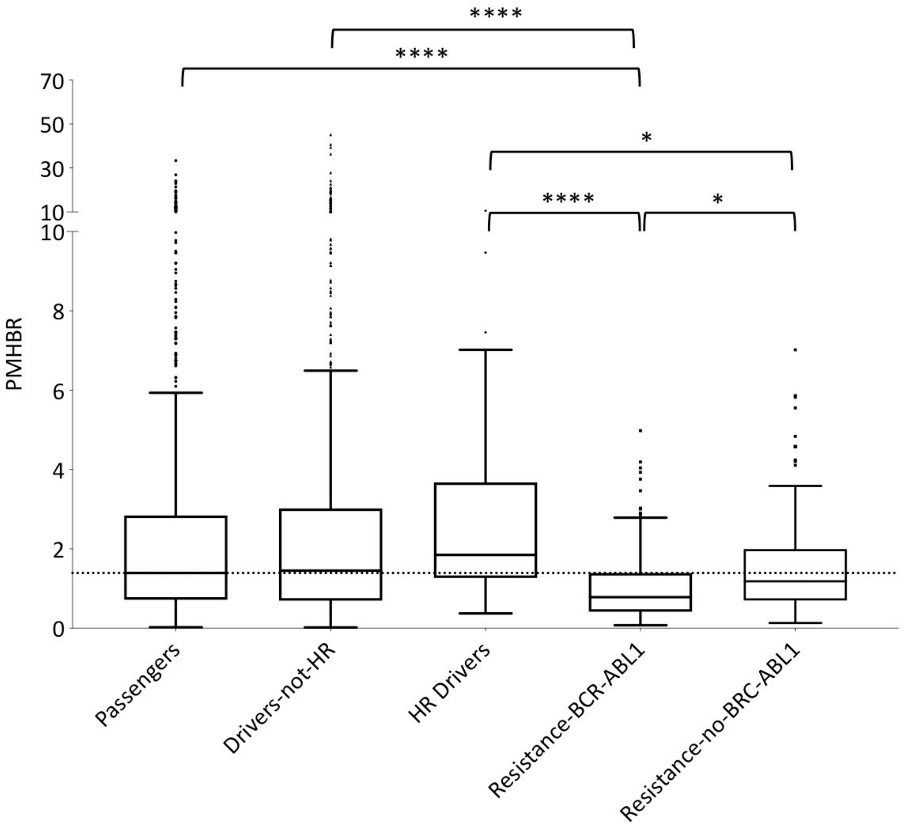

Supplementary Figure 9. *Similar to Figure 1 but splitting the resistance mutation dataset into mutations found in the BCR-ABL1 fusion gene versus other genes instead of by recurrence.* Note that, for the sake of readability, the part of the y-axis corresponding to values of PMHBR above 10 is compressed. The dotted horizontal line is a guide for the eye and corresponds to the value of the median of the distribution for passenger mutations. Asterisks indicate significance of differences between PMHBR score distributions calculated using a Kruskal-Wallis test followed by Dunn's *post hoc* test. p-values are adjusted for multiple testing (all vs all). (*) stands for p-value<0.05,and (****) for p-value<0.0001.

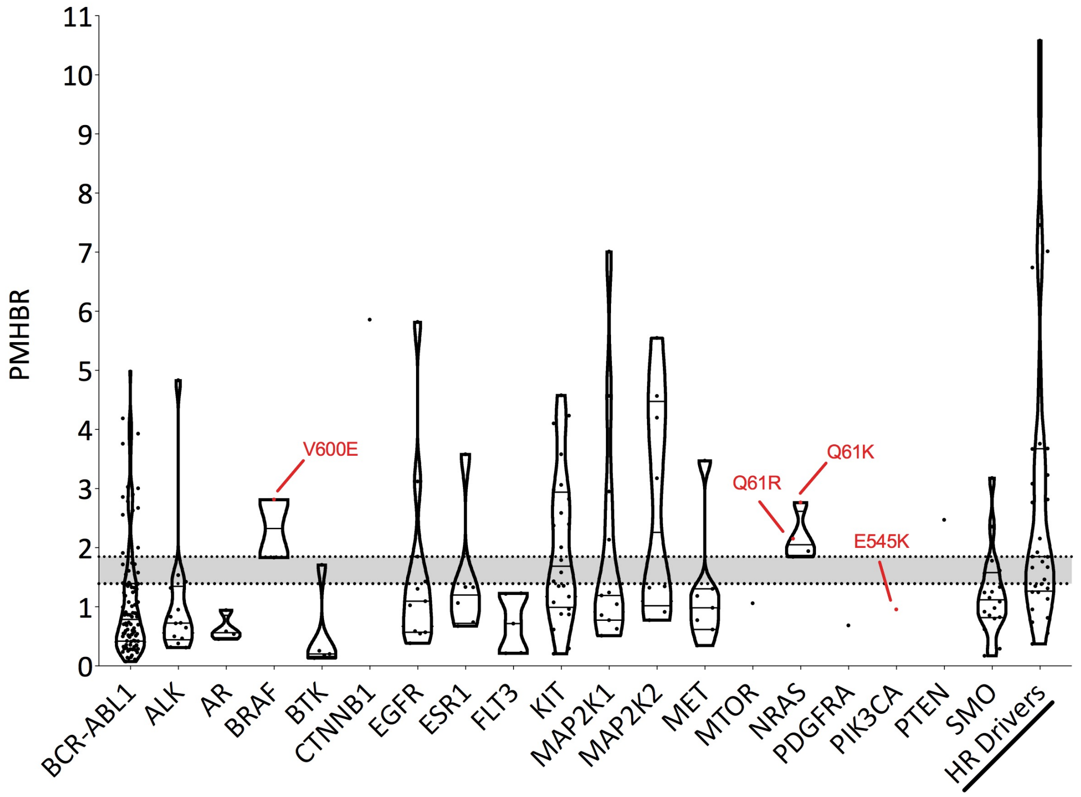

Supplementary Figure 10. *Violin plots of the distributions of PMHBR scores for 19 genes in our COSMIC resistance mutation dataset and for HR Driver mutations.* Lower PMHBR values correspond to a higher likelihood of being presented by HLA class I complexes. In red, we highlighted and labelled the four resistance mutations that are also part of the HR Drivers set. The dotted horizontal lines are guides for the eye and correspond to the value of the median of the distribution for passenger (bottom) and HR Driver mutations (top), respectively (as seen for example in Figure 1).

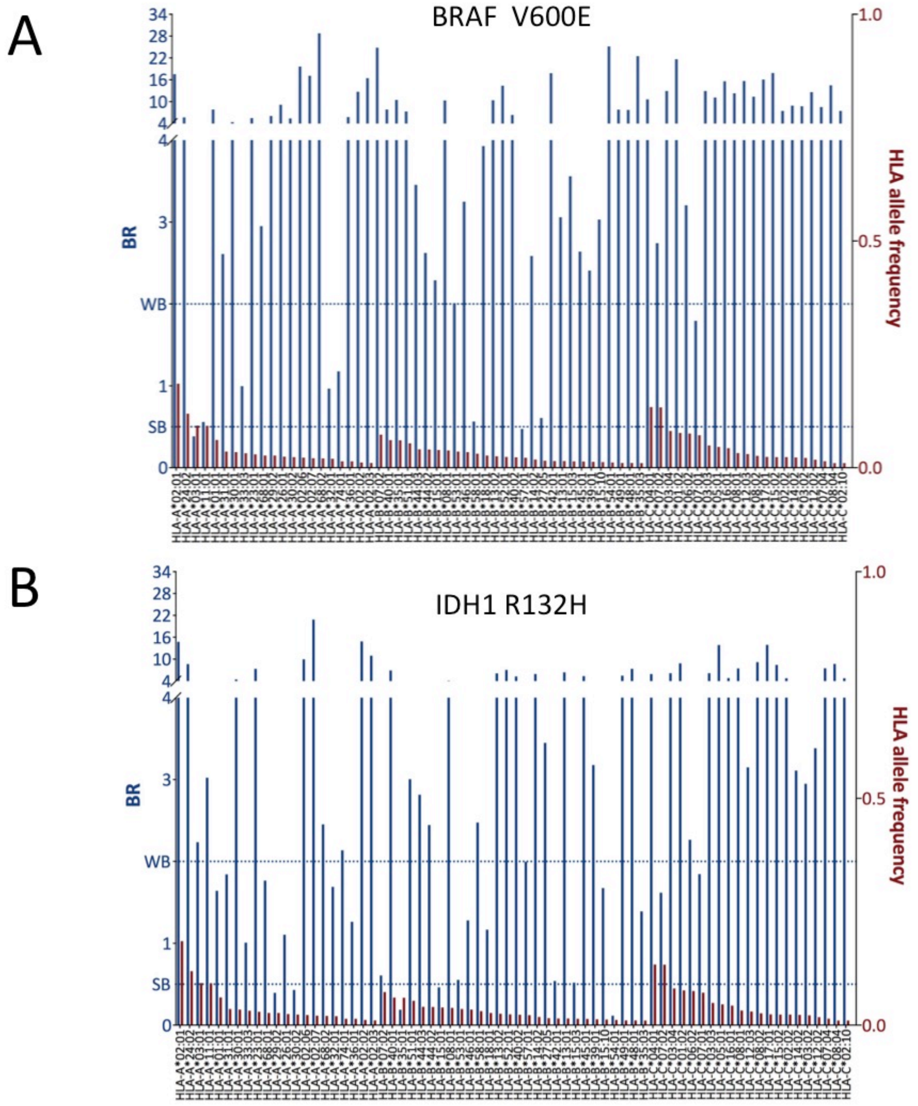

Supplementary Figure 11. *BR score HLA-presentation profiles of two common somatic mutations (most common in our TCGA dataset): **A)** V600E in BRAF (present in 561 TCGA patients) and **B)** R132H in IDH1 (384 TCGA patients).* Blue bars (primary y-axis) represent the BR scores of the mutation with respect to the HLA alleles reported on the x-axis. Red bars (secondary y-axis) represent the frequency of each HLA allotype in the 1000G dataset. We report BR scores for only the HLA-A, -B and -C allotypes that have frequency >1%. The two dotted lines mark elution rank value limits for strong likelihood of presentation (SB, i.e score < 0.5) and weaker likelihood of presentation (WB, i.e. 0.5<score<2.0). Note that for the sake of readability the part of the primary y-axis corresponding to values of BR above 4 is compressed.

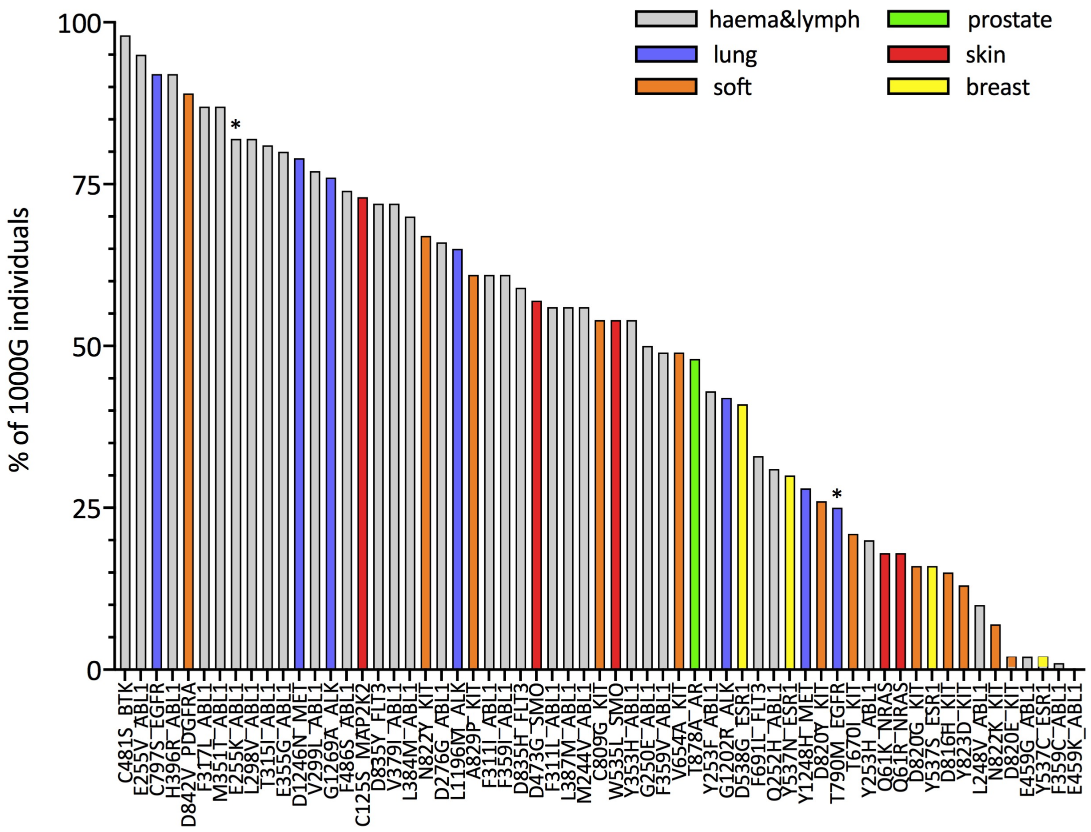

Supplementary Figure 12. *Equivalent to Figure 3 when using only HLA-A and HLA-B for calculating the IBR score..*

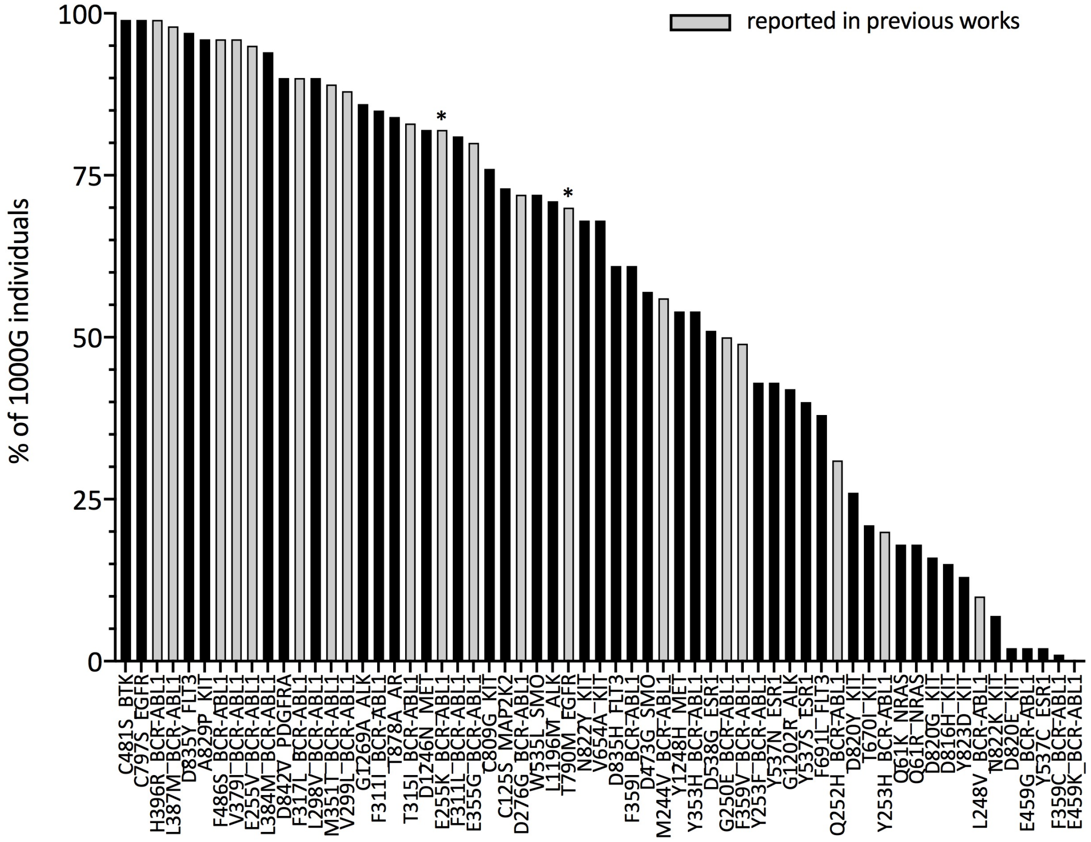

Supplementary Figure 13. *Same as Figure 3 with the only difference that instead of coloring the histogram's bars according to the tumor tissues the mutations occur in, we highlight in light grey what (to our knowledge) are all mutations that have been studied* in silico *in previous works for their likelihood to generate neopeptides that are HLA-presented* (Cai, A. *et al.* Mutated BCR-ABL generates immunogenic T-cell epitopes in CML patients. *Clin Cancer Res* **18**, 5761-5772, doi:10.1158/1078-0432.CCR-12-1182 (2012); Yamada, T. *et al.* EGFR T790M mutation as a possible target for immunotherapy; identification of HLA-A*0201-restricted T cell epitopes derived from the EGFR T790M mutation. *PLoS One* **8**, e78389, doi:10.1371/journal.pone.0078389 (2013); Ofuji, K. *et al.* A peptide antigen derived from EGFR T790M is immunogenic in nonsmall cell lung cancer. *Int J Oncol* **46**, 497-504, doi:10.3892/ijo.2014.2787 (2015)). As in Figure 3, asterisks indicate what mutations in those works have further been tested *in vitro* and successfully shown to generate neopeptides that are able to elicit T-cell responses. Note that absence of asterisks from most of the previously studied mutations does not indicate that they cannot elicit T-cell responses but rather that they are not known to have been tested for it.

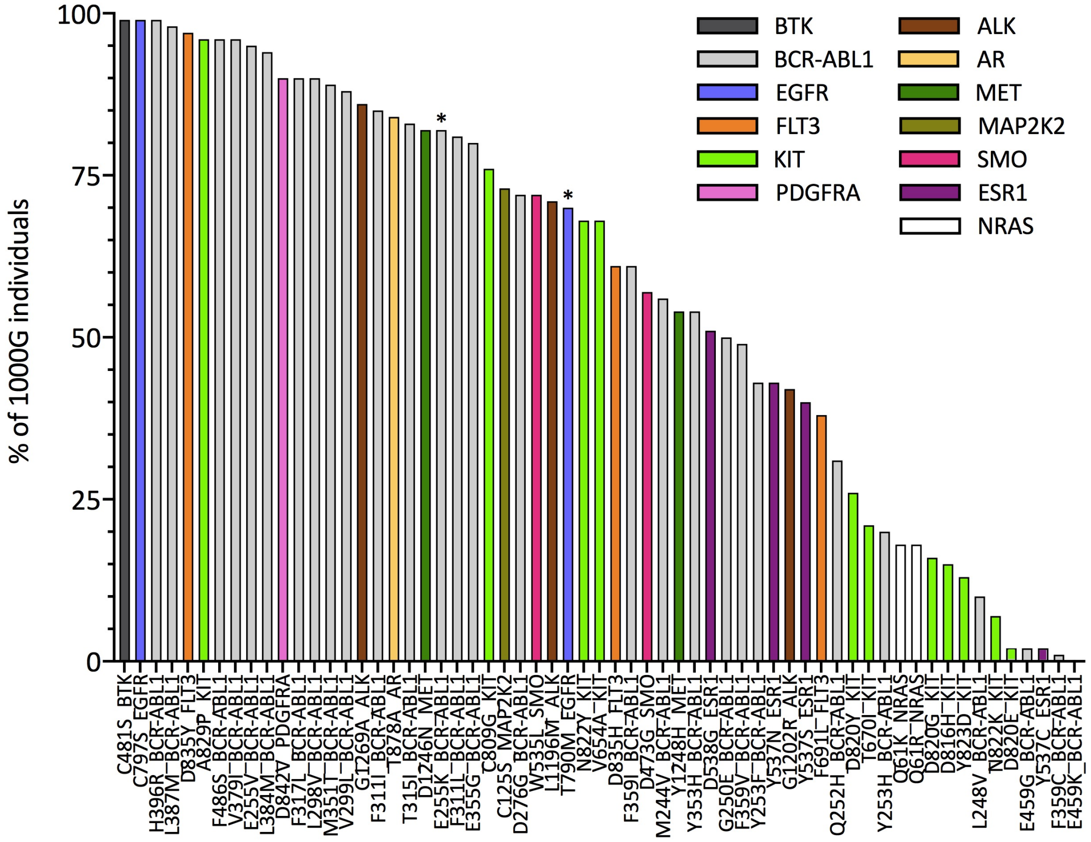

Supplementary Figure 14. *Same as Figure 3 with the only difference that histogram bars' colors indicate the different genes in which the mutations are found rather than the tumor tissues.*

Q

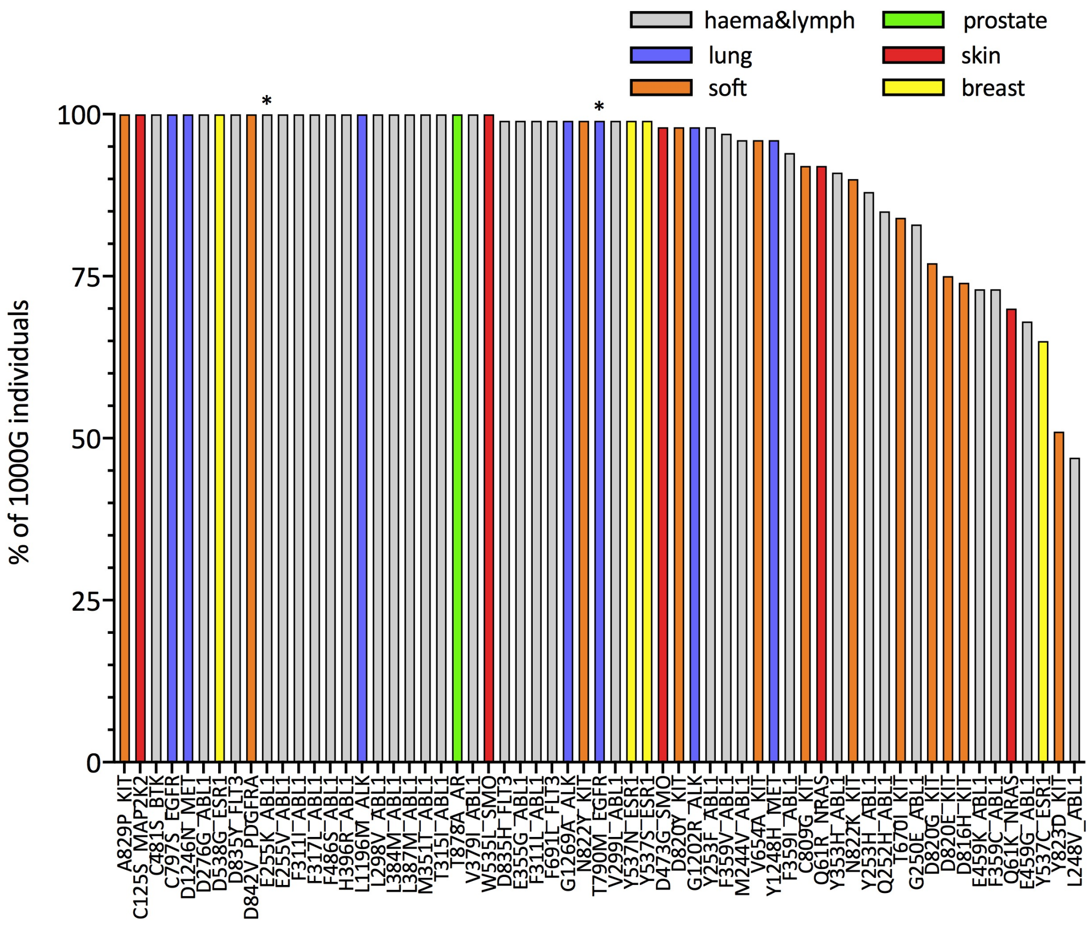

Supplementary Figure 15. *Equivalent of Figure 3 when considering a less restrictive IBR threshold (IBR<2.0).*

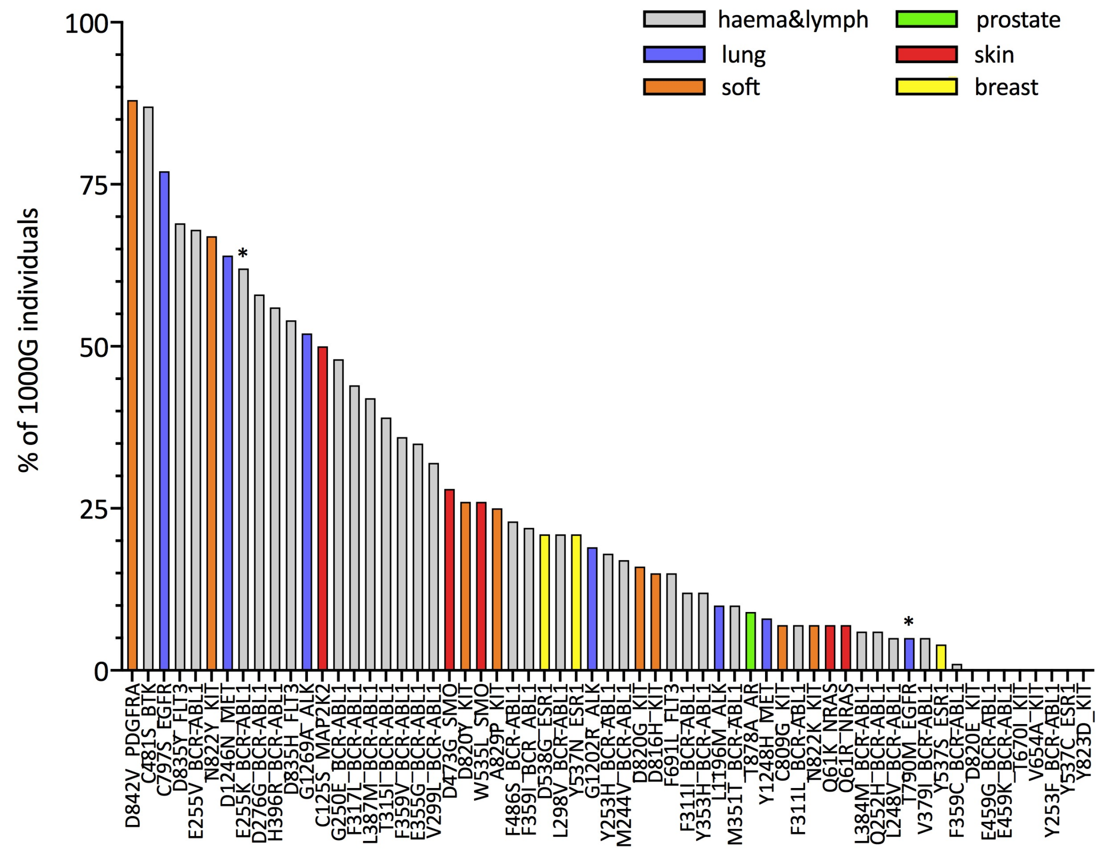

Supplementary Figure 16. *Equivalent to Figure 4 when using only HLA-A and HLA-B for calculating* the MinRank of the mutant and corresponding wild type peptides.

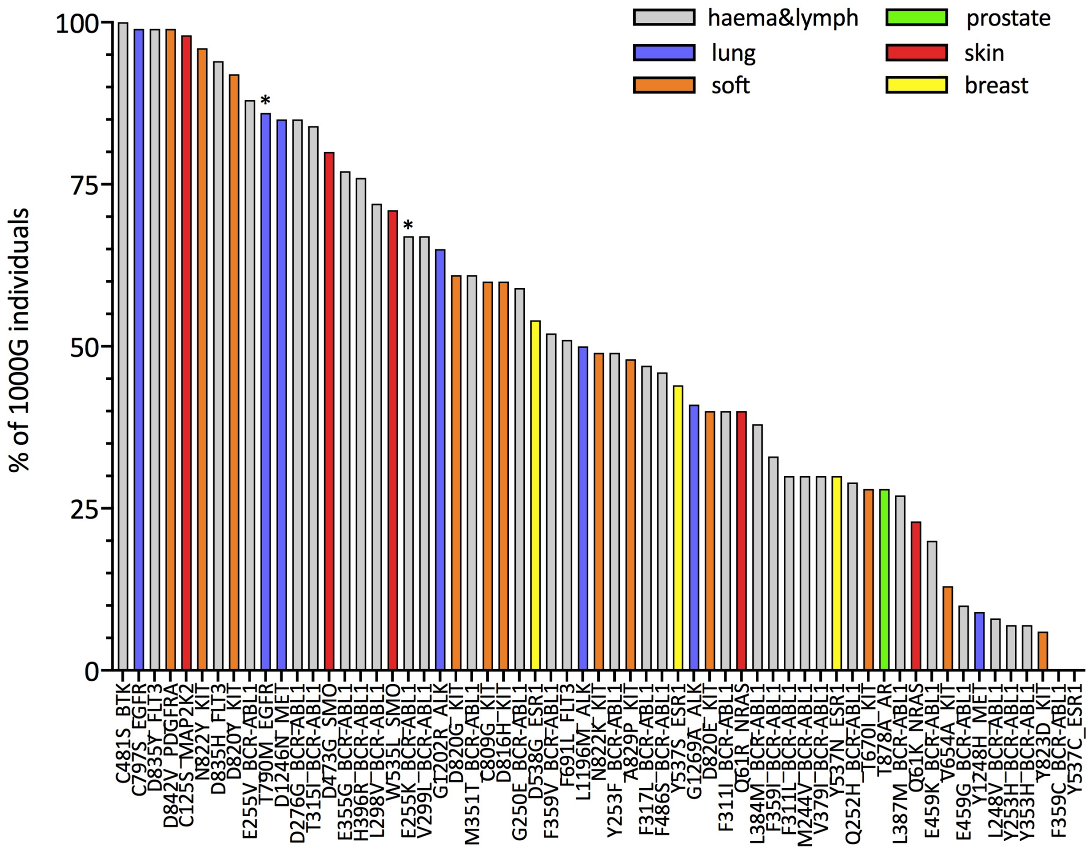

Supplementary Figure 17. *Equivalent to Figure 4 but using 2.0 as MinRank threshold.* For each mutation, the histogram illustrates the estimated percentage of individuals for which at least one mutant peptide-wild type peptide pair exists such that the MinRank of the mutant peptide is <2.0 and the MinRank of the corresponding wild type peptide is ≥2.0 (see **Methods** for definitions). Mutations on the x-axis are ordered according to decreasing percentages of individuals. We plot only mutations that have been observed in at least 5 patients (according to COSMIC). Colours indicate the different tumour tissues in which the resistance mutations have been observed; “haema&lymph” stands for haematopoietic and lymphoid tissue. Asterisks (*) mark mutations that have been shown to elicit T-cell responses in previous works.

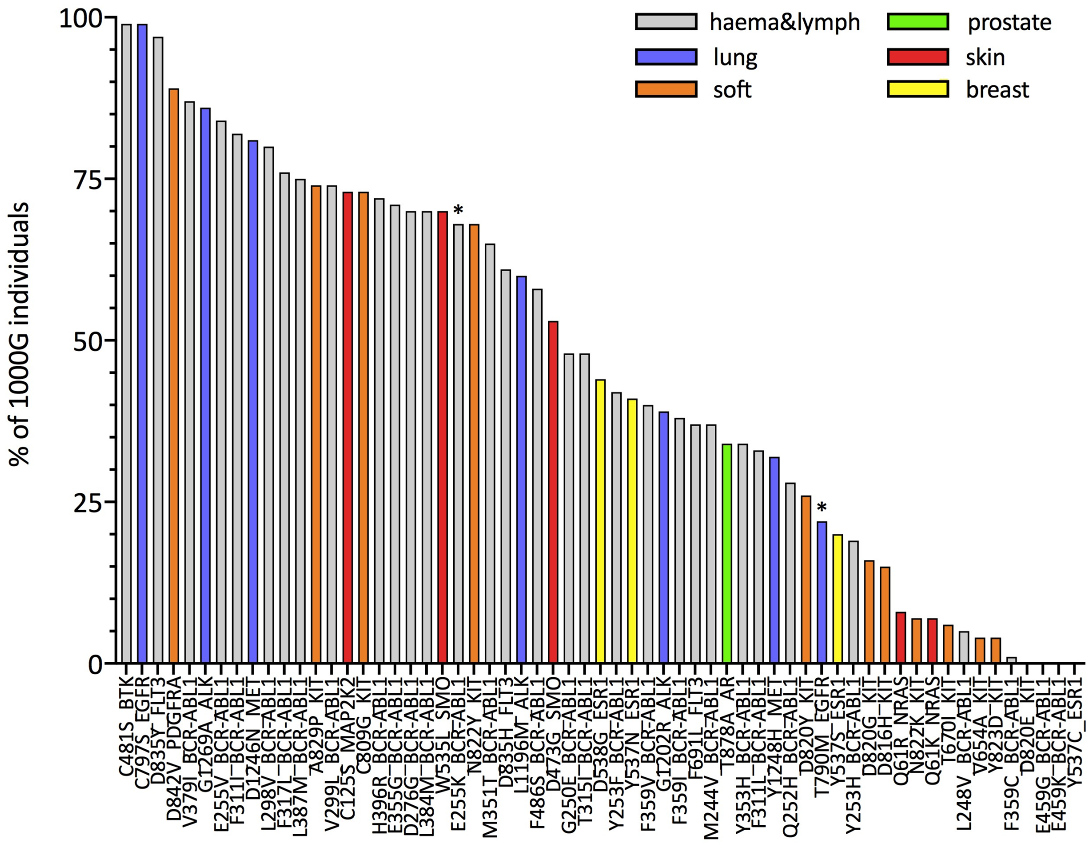

Supplementary Figure 18. *Equivalent to Figure 4 but asking that mutant peptides have MinRank<0.5 and lower than corresponding wild type peptides.* In other words, for each mutation, the histogram illustrates the estimated percentage of individuals for which there exists at least one mutant peptide-wild type peptide pair such that the MinRank of the mutant peptide is <0.5 and the MinRank of the corresponding wild type peptide is higher than that of the mutant peptide (note: could still be <0.5). Mutations on the x-axis are ordered according to decreasing percentages of individuals. We plot only mutations that have been observed in at least 5 patients (according to COSMIC). Colours indicate the different tumour tissues in which the resistance mutations have been observed; "haema&lymph" stands for haematopoietic and lymphoid tissue. Asterisks (*) mark mutations that have been shown to elicit T-cell responses in previous works.

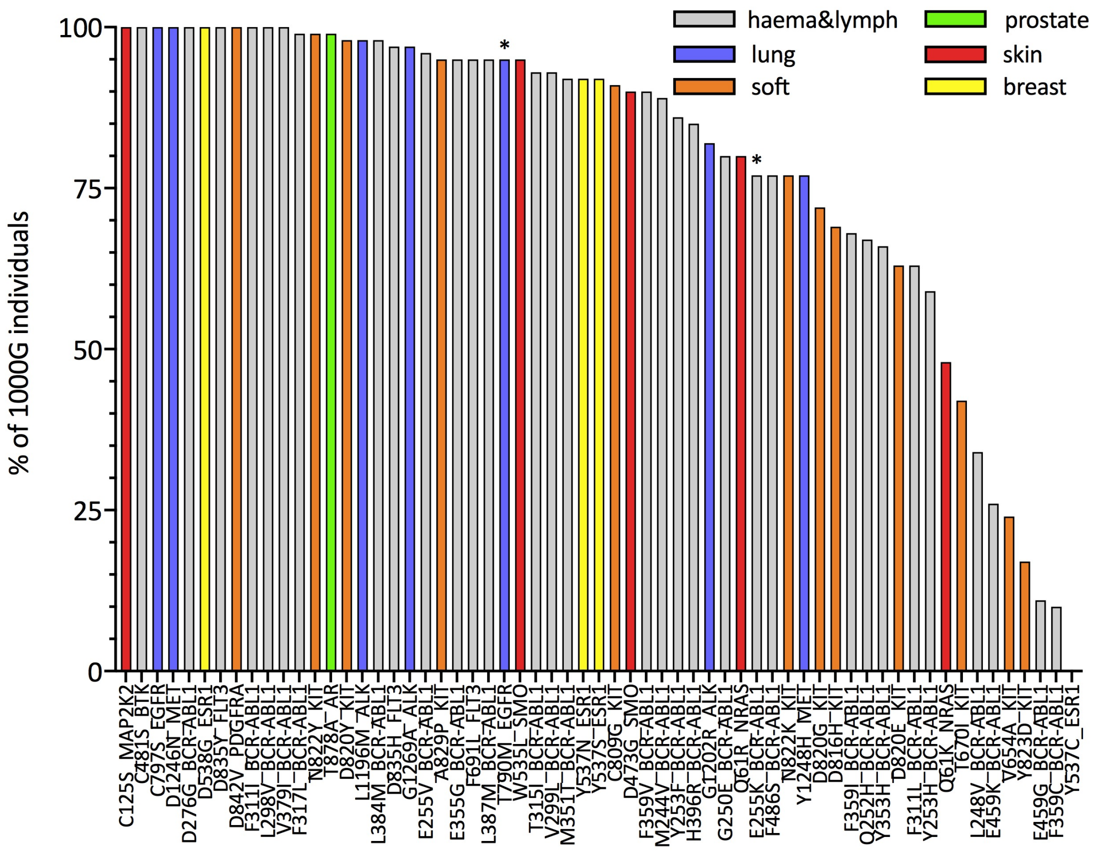

Supplementary Figure 19. *Equivalent to Supplementary Figure 18 but using 2.0 as MinRank threshold.*

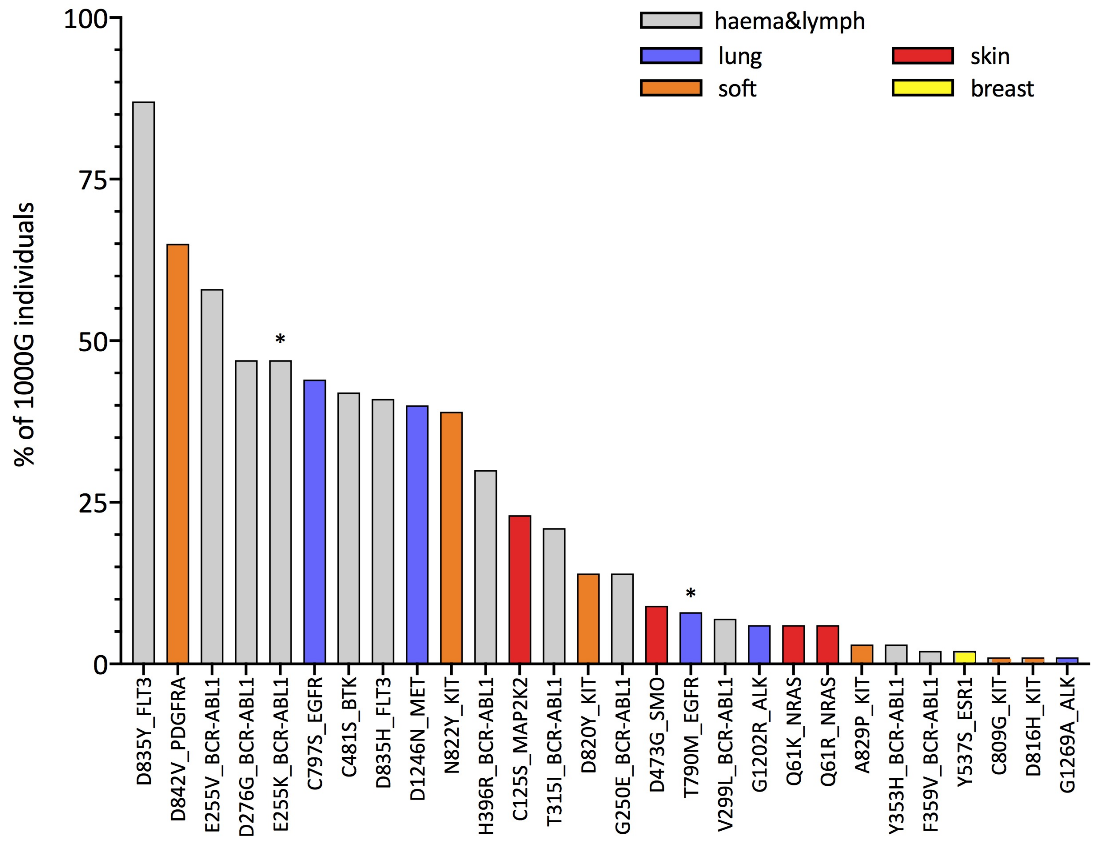

Supplementary Figure 20. *Equivalent to Figure 4 but asking that mutant peptides have MinRank <0.5 and corresponding wild-type peptides have MinRank >2.0.* This means that, for each mutation, the histogram illustrates the estimated percentage of individuals for which there exists at least one mutant-wild type peptide pair such that the MinRank of the mutant peptide is <0.5 and the MinRank of the corresponding wild type peptide is >2.0. Mutations on the x-axis are ordered according to decreasing percentages of individuals. We plot only mutations that have been observed in at least 5 patients (according to COSMIC) and for which the percentage of individuals is at least 1. Colours indicate the different tumour tissues in which the resistance mutations have been observed; "haema&lymph" stands for haematopoietic and lymphoid tissue. Asterisks (*) mark mutations that have been shown to elicit T-cell responses in previous works.

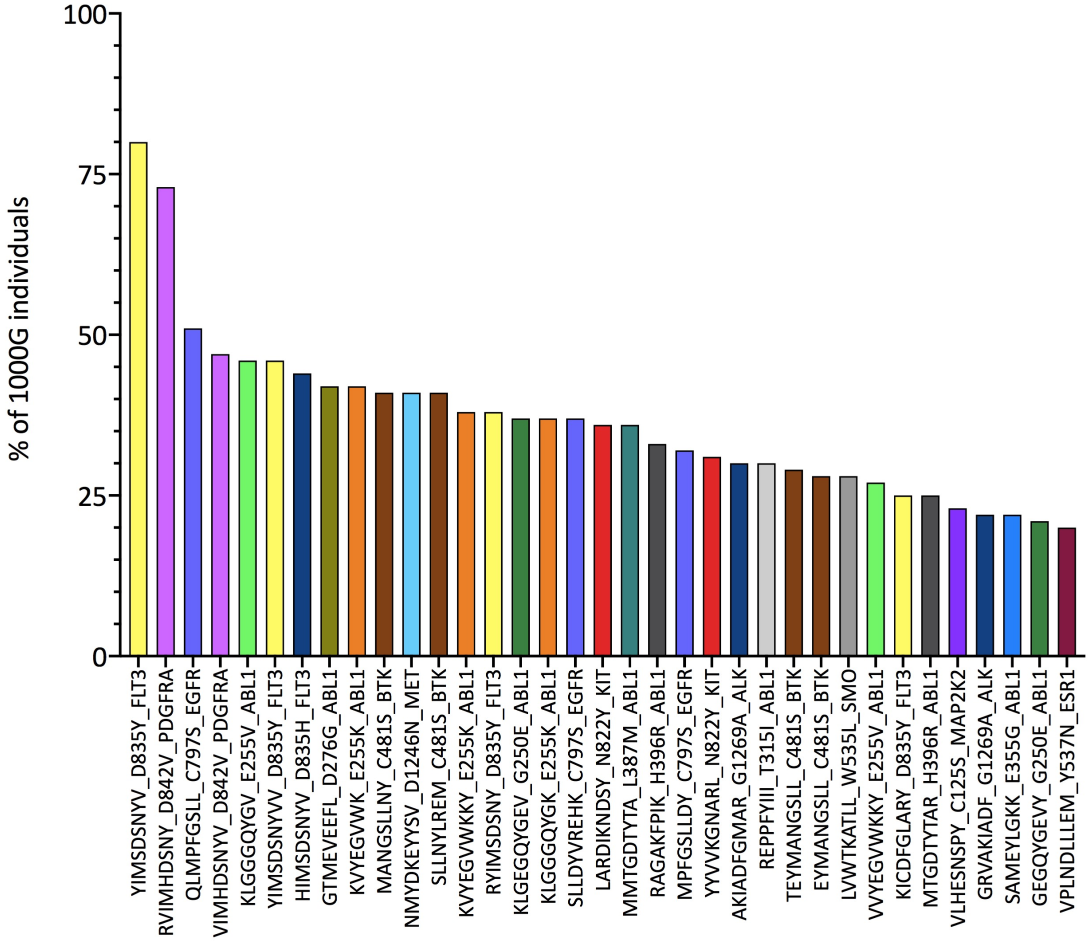

Supplementary Figure 21. *Estimates of the percentage of individuals in the general population for which a specific mutant peptide is predicted more likely to be presented with respect to its wild-type counterpart.* More specifically, the histogram illustrates the estimated percentage of individuals in which a given mutant peptide has MinRank<0.5 while the MinRank for its corresponding wild type peptide is >0.5. Mutant peptides on the x-axis are ordered according to decreasing percentages of individuals. We consider only mutant peptides from resistance mutations that occur in at least 5 patients in COSMIC and, for clarity, we plot only mutant peptides with the percentage of individuals satisfying the above conditions being >=20%. Colours indicate peptides associated to different mutations.

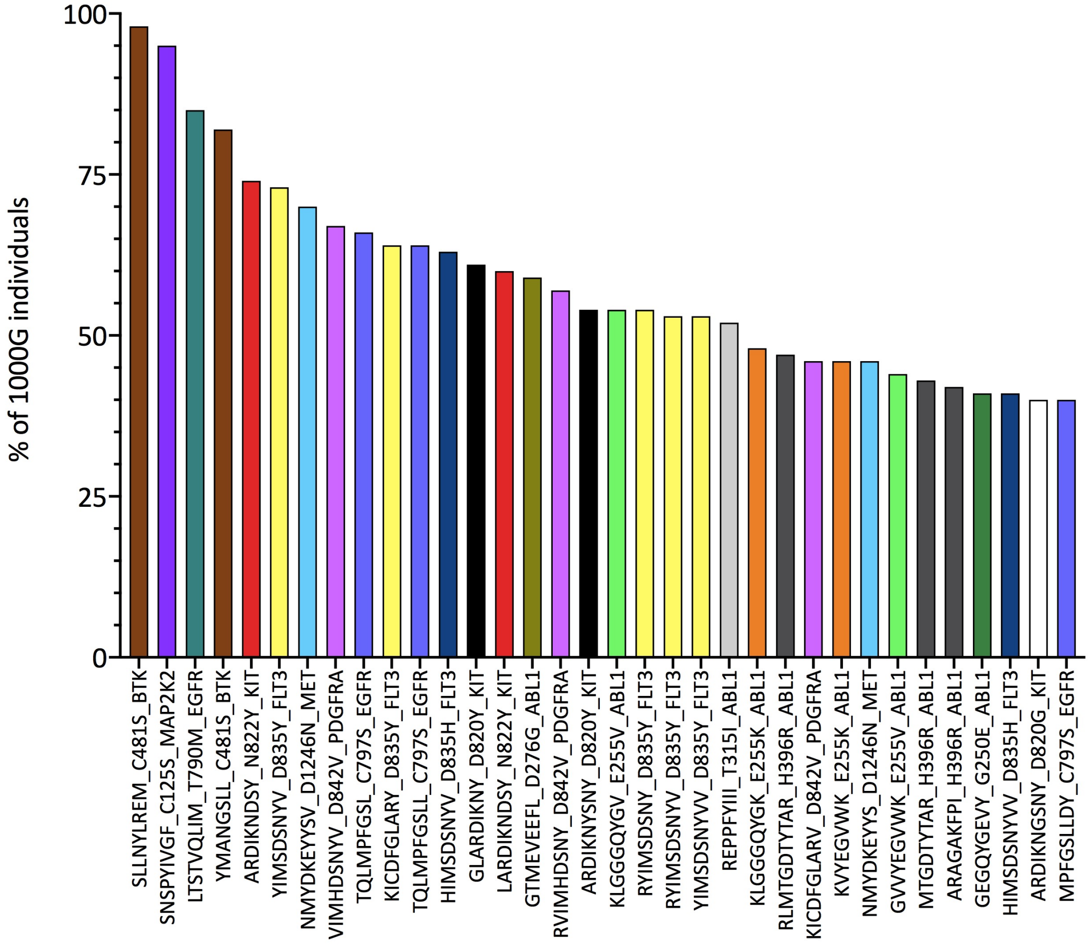

Supplementary Figure 22. *Equivalent to Supplementary Figure 16 but using a MinRank of 2.0 instead of 0.5.* We consider only mutant peptides from resistance mutations that occur in at least 5 patients in COSMIC and, for clarity, we plot only mutant peptides with the percentage of individuals satisfying the above conditions being >=40%. Colours indicate peptides associated to different mutations.

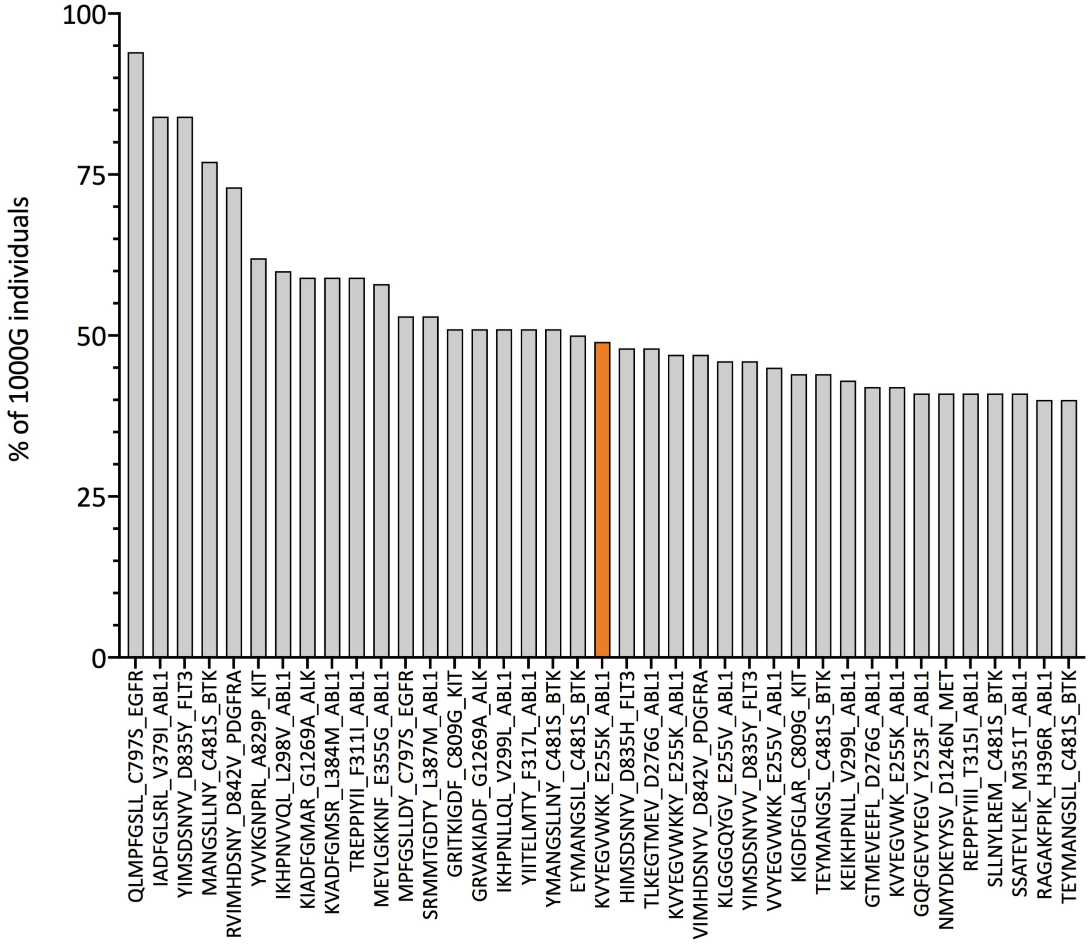

Supplementary Figure 23. *Equivalent to Supplementary Figure 21 but asking that mutant peptides have MinRank<0.5 and lower than corresponding wild type peptides.* More specifically, the histogram illustrates the estimated percentage of individuals in which a given mutant peptide has a MinRank<0.5 and the MinRank of the corresponding wild type peptide is higher than that of the mutant peptide (note: could still be <0.5). Mutant peptides on the x-axis are ordered according to decreasing percentages of individuals. We consider only mutant peptides from resistance mutations that occur in at least 5 patients in COSMIC and, for clarity, we plot only mutant peptides with the percentage of individuals satisfying the above conditions being >=40. The orange bar highlights one of the peptides that in a previous study was shown *in vitro* to be able to elicit T-cell responses (Cai, A. *et al.* Mutated BCR-ABL generates immunogenic T-cell epitopes in CML patients. *Clin Cancer Res* **18**, 5761-5772, doi:10.1158/1078-0432.CCR-12-1182 (2012)).

BCR-ABL1 Imatinib resistance mutations

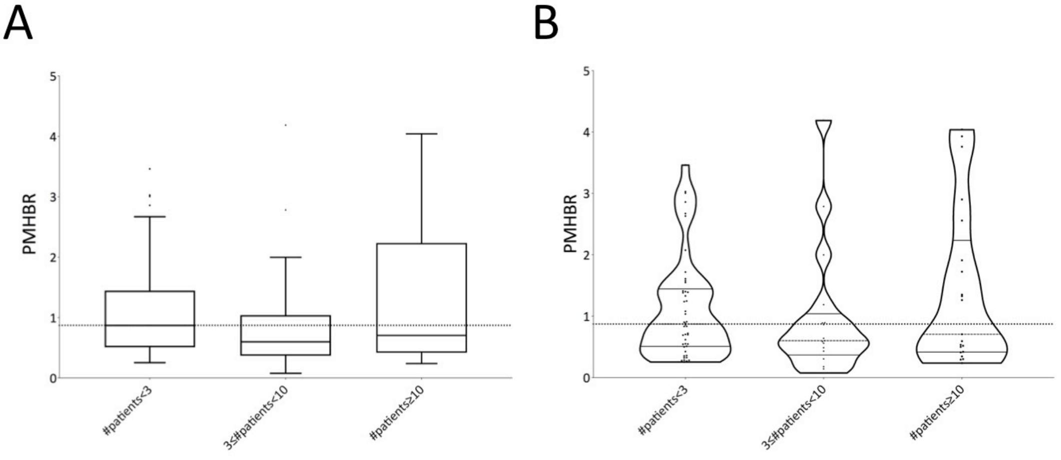

Supplementary Figure 24. A) *Box plots and* ***B)*** *violin plots of the distribution of PMHBR scores for 3 sets of BCR-ABL1 mutations that have been reported to confer resistance to the drug imatinib*. The different sets are characterised by a different number of patients in which the mutations have been observed (according to COSMIC). Lower PMHBR values correspond to a higher likelihood of being presented by HLA class I complexes. The dotted horizontal line is a guide for the eye and corresponds to the value of the median of the distribution for mutations that have been observed in less than 3 patients. The lower and higher edges of each Tukey box represent the 25% and 75% percentile value, respectively. The horizontal line inside each box (violin) represents the median value. Horizontal lines within each violin plot indicate 25th percentile, median and 75th percentile (bottom, middle and top, respectively). Observed differences are not significant (Kruskal-Wallis test followed by Dunn's *post hoc* test).

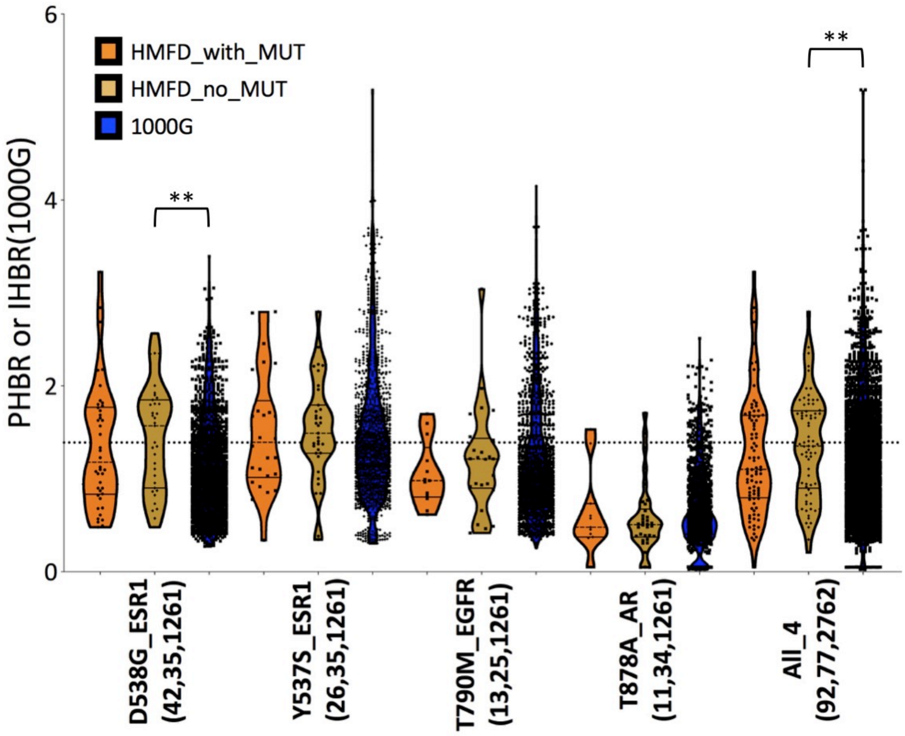

Supplementary Figure 25. *Same as Figure 5 but using violin plots and staggered dots.* Lower PHBR values correspond to a higher likelihood of being presented by HLA class I complexes. For each mutation, "HMFD_with_MUT" and "HMFD_no_MUT" represent patients treated with similar targeted drugs, however, "HMFD_with_MUT" (orange) are patients that developed the mutation while "HMFD_no_MUT" (yellow) are patients that did not develop it. "1000G" (blue) are all the healthy individuals in our 1000G dataset. On the x-axis, for each mutation we report in parentheses the number of patients/individuals considered in each of the three groups (the order is "HMFD_with_MUT", "HMFD_no_MUT", "1000G"). Note that when considering all four mutations together, for a fair comparison, "no_MUT" patients and "1000G" healthy individuals have been sampled so to obtain a proportion of scores derived from each mutation similar to the one observed among "with_MUT" patients. "HMFD_no_MUT" for ESR1 D538G and Y537S are the same group of breast cancer patients. Asterisks indicate significance of pairwise differences between score distributions calculated using Mann-Whitney tests with no multiple comparison adjustment. (**) stands for p-value < 0.01. Pairwise tests were performed only between distributions in the same mutation group. The lower and higher edges of each Tukey box represent the 25% and 75% percentile value, respectively. The horizontal line inside each box represents the median value.

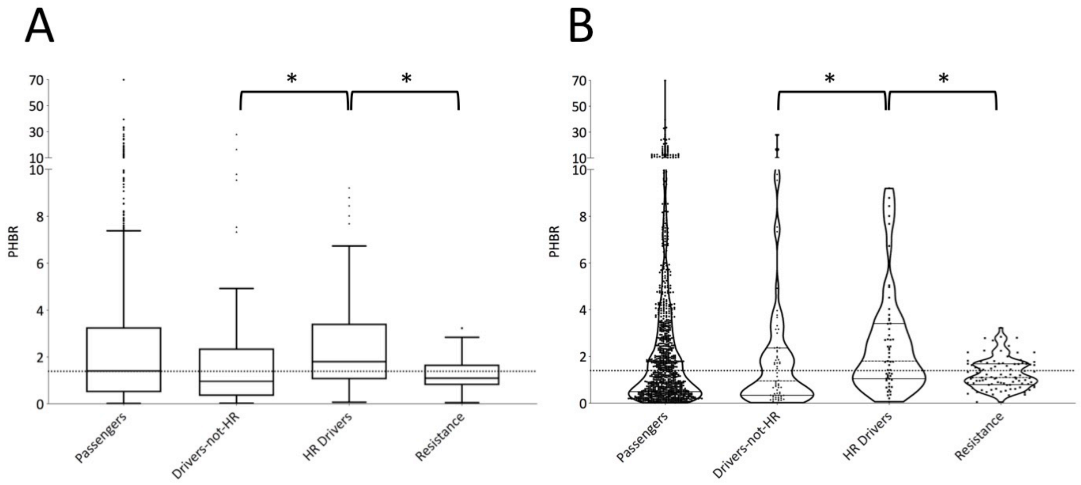

Supplementary Figure 26. *Distribution of PHBR scores for different sets of mutations in a subset of HMFD patients, shown as either box plots **A)** or violin plots with staggered dots **B).*** We consider passenger and driver mutations (**Methods**) as well as the four resistance mutations analysed in details in Figures 5 and 6. The distribution of driver mutation scores is split according to whether or not they overlap with the list of HR drivers in the TCGA database. Lower PHBR values correspond to a higher likelihood of being presented by HLA class I complexes. The dotted horizontal line is a guide for the eye and corresponds to the value of the median of the distribution for passenger mutations. Note that, for the sake of readability, the part of the y-axis corresponding to values of PMHBR above 10 is compressed. The lower and higher edges of each Tukey box represent the 25% and 75% percentile value, respectively. The horizontal line inside each box represents the median value. Asterisks indicate significance of differences between PMHBR score distributions calculated using a Kruskal-Wallis test followed by Dunn's *post hoc* test. p-values are adjusted for multiple testing (all vs all). (*) stands for p-value<0.05. Note that the Resistance dataset contains PHBR scores from only 4 different resistance mutations (see Text), thus it cannot be considered representative for the distribution of the wider set of resistance mutations in COSMIC.

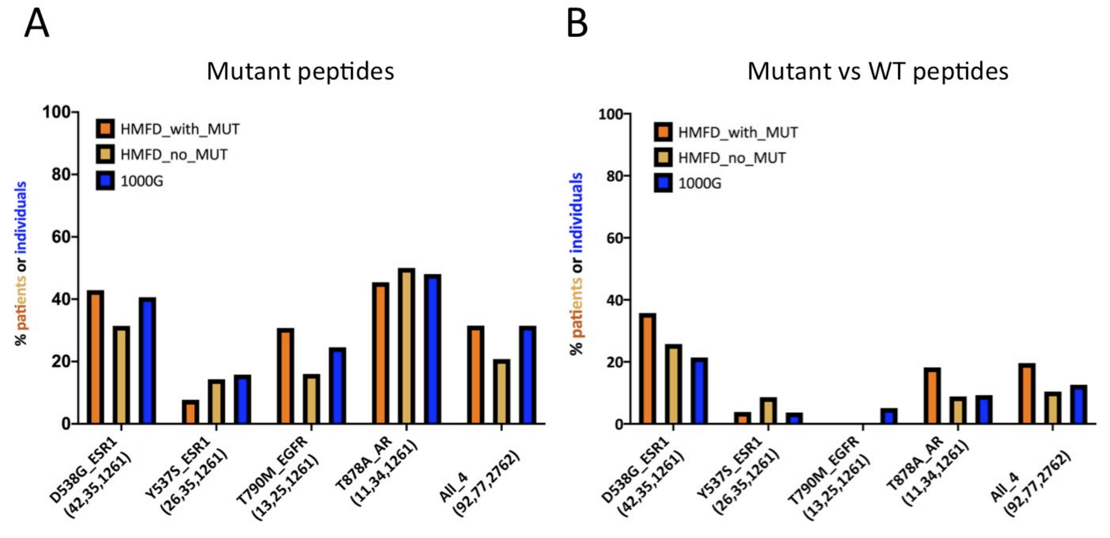

Supplementary Figure 27. *Equivalent to Figure 6 when using only HLA-A and HLA-B for calculating the PBR, IBR* ***A)*** *and MinRank* ***B)*** *scores.* **A)** *Estimates for the percentage of patients or individuals predicted to HLA-present neopeptides associated to four different resistance mutations.* Groups of patients and of healthy individuals as well as notations are as described in Figure 5. For each mutation in each group, the histogram illustrates the percentage of patients with a PBR<0.5 (HMFD patients) or IBR<0.5 (1000G). Both PBR and IBR scores are defined in **Methods**. **B)** *Comparison between mutant peptides and corresponding wild type peptides for the same mutations and groups of patients/individuals shown in panel (A).* For each mutation and each group, the histogram illustrates the estimated percentage of patients or healthy individuals for which at least one mutant-wild type peptide pair exists such that the MinRank of the mutant peptide is <0.5 and the MinRank of the corresponding wild type peptide is ≥0.5 (see **Methods** for definitions). Estimates in (A) and (B) for 1000G are the same as those reported for these mutations in Supplementary Figures 12 and 16, respectively.

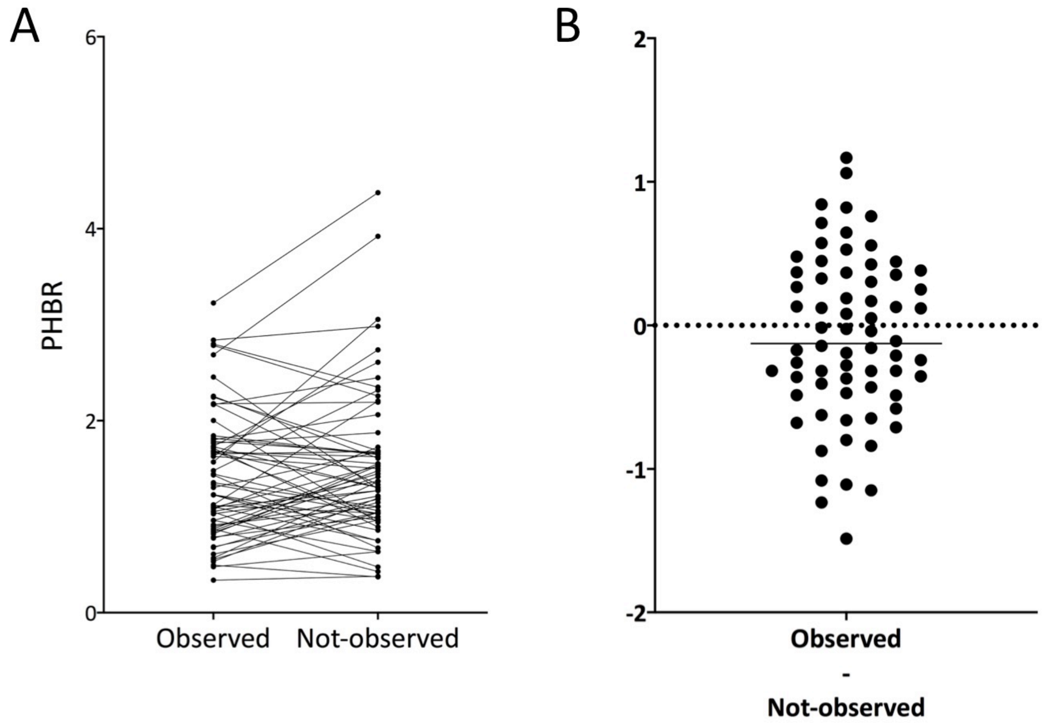

Supplementary Figure 28. Comparison between the PHBR scores of the D538G ESR1 and Y537S ESR1 mutations in the same HMFD patients. Each of the 68 patients considered has either one mutation (42 have D538G) or the other (26 have Y537S). The set of "Observed" values represents the PHBR score of the mutations that occur in the respective patients. These are paired with the PHBR mutation that is "Not-observed" in the same patient. **A)** PHBR scores for "Observed" and "Not-observed" mutations. Scores referring for the same patient are connected by a line. **B)** Plot of the differences between the paired "Observed" and "Not-observed" mutation scores shown in A). The difference between paired scores is not significant (Wilcoxon test). The difference is not significant also when considering a balanced set of patients carrying the mutations (that is, 26 patients for each mutation, not shown).

Supplementary Tables

Supplementary Table 1. *Lists of TCGA and COSMIC mutations analysed in this manuscript and of their associated PMHBR scores.*

Supplementary Table 2. *List of resistance mutations analysed in the manuscript along with data regarding the number of patients they have been reported in, the tissues, tumor subtypes and drugs they relate to (source: COSMIC version 86)*

Supplementary Table 3. *Mapping between EnsEMBL transcript IDs and gene names (HGNC symbols) used when creating the final lists of TCGA passengers, drivers, germline SNPs and random mutations.*

Supplementary Table 4. *List of HLA class I allotype combinations for 1,261 individuals in the 1000 Genomes Project (Gourraud, P. A. et al. HLA diversity in the 1000 genomes dataset. PLoS One* ***9****, e97282, doi:10.1371/journal.pone.0097282 (2014)).*

Supplementary Table 5. *Lists of HMFD samples analysed in this manuscript and of their predicted HLA class I allotype combinations and PHBR scores for all mutations datasets shown in Figure 5 and Supplementary Figure 28. Note that data in the "PHBR ESR1 paired muts –balanced" sheet have been balanced using random sampling without replacement.*

Supplementary Table 6. *Lists of mutations analysed in this manuscript and of their associated PMHBR scores calculated using HLA-A and HLA-B only to calculate the score or all three HLA genes but TCGA patients' HLA class I allotype combinations instead of 1000G's.*

Supplementary Table 7. Tab "Figure 3 – all mutations". *Percentage of the 1000G individuals (Supplementary Table 4) that are estimated to HLA-present at least one of the resistance mutation-associated neopeptides when using an IBR score threshold of <0.5.* **Tab "Figure 4 – all mutations".** *Percentage of 1000G individuals in which at least one mutant peptide is predicted highly likely to be presented (MinRank<0.5) while the corresponding wild type peptide is not highly likely to be presented (MinRank≥0.5).*

Supplementary Table 8. *Lists of resistance mutation-associated neopeptides that are more likely to be HLA-presented than their wild type counterparts when considering three different sets of thresholds: mutant peptide MinRank <0.5 and wild type peptide MinRank>0.5, mutant peptide MinRank <2.0 and wild type peptide MinRank>2.0 and, finally, mutant peptide MinRank<0.5 and wild type peptide MinRank>mutant peptide MinRank. Only peptides associated to resistance mutations observed in at least 5 patients (COSMIC version 86) and predicted to be presented by at least 1% of individuals are shown.*
